# BCL9 provides multi-cellular communication properties in colorectal cancer by interacting with paraspeckle proteins

**DOI:** 10.1038/s41467-019-13842-7

**Published:** 2020-01-07

**Authors:** Meng Jiang, Yue Kang, Tomasz Sewastianik, Jiao Wang, Helen Tanton, Keith Alder, Peter Dennis, Yu Xin, Zhongqiu Wang, Ruiyang Liu, Mengyun Zhang, Ying Huang, Massimo Loda, Amitabh Srivastava, Runsheng Chen, Ming Liu, Ruben D. Carrasco

**Affiliations:** 1 Department of Oncologic Pathology, Dana-Farber Cancer Institute, Harvard Medical School, Boston, MA 02115 USA; 2 Department of General Surgery, Fourth Affiliated Hospital of Harbin Medical University, Harbin Medical University, Harbin, 150001 China; 3 Key Laboratory of RNA Biology, Institute of Biophysics, Chinese Academy of Sciences, Beijing, 100101 China; 4 Department of Experimental Hematology, Institute of Hematology and Transfusion Medicine, Warsaw, 02776 Poland; 5 Department of Obstetrics and Gynecology, Fourth Affiliated Hospital of Harbin Medical University, Harbin Medical University, Harbin, 150001 China; 6 Depatment of Radiation Oncology and Cyberknife Center, Key Laboratory of Cancer Prevention and Therapy, Tianjin Medical University Cancer Institute & Hospital, National Clinical Research Center for Cancer, Tianjin, 300060 China; 7 Department of Pathology, Brigham and Women’s Hospital, Harvard Medical School, Boston, MA 02115 USA

**Keywords:** Cancer, Colorectal cancer, Cell biology, Molecular biology

## Abstract

Colorectal cancer (CRC) is the third most commonly diagnosed cancer, which despite recent advances in treatment, remains incurable due to molecular heterogeneity of tumor cells. The B-cell lymphoma 9 (BCL9) oncogene functions as a transcriptional co-activator of the Wnt/β-catenin pathway, which plays critical roles in CRC pathogenesis. Here we have identified a β-catenin-independent function of BCL9 in a poor-prognosis subtype of CRC tumors characterized by expression of stromal and neural associated genes. In response to spontaneous calcium transients or cellular stress, BCL9 is recruited adjacent to the interchromosomal regions, where it stabilizes the mRNA of calcium signaling and neural associated genes by interacting with paraspeckle proteins. BCL9 subsequently promotes tumor progression and remodeling of the tumor microenvironment (TME) by sustaining the calcium transients and neurotransmitter-dependent communication among CRC cells. These data provide additional insights into the role of BCL9 in tumor pathogenesis and point towards additional avenues for therapeutic intervention.

## Abstract (web summary)

BCL9 is an activator of oncogenic Wnt/β-catenin. Here, the authors show a β-catenin independent function of BCL9 in a subtype of colorectal cancers, where it interacts with paraspeckle proteins to enhance the mRNA stability of calcium signalling and neural associated genes to promote communication with tumour cells and its microenvironment.

## Introduction

The human *BCL9* gene, a homolog of the *Drosophila* segment polarity gene *Legless* was first identified in a (1;14)(q21;q32) translocation from a patient with precursor B-cell acute lymphoblastic leukemia (B-ALL)^1^. BCL9/Legless is a transcriptional co-activator of the canonical Wnt pathway and bind to β-catenin through a highly conserved HD2 domain (BCL9-HD2)^2–5^. The oncogenic potential of *BCL9* in human cancer is further highlighted by studies showing that: (i) chromosome 1q21 amplifications harboring the *BCL9* locus are observed in a broad range of cancers and are associated with poor clinical outcome^6,7^; (ii) *BCL9* is upregulated in various malignancies as a consequence of downregulation of microRNAs^7–12^ that function as endogenous tumor suppressors of *BCL9*; (iii) LATS2^13^ and SOX7^14^ proteins, which suppress oncogenic Wnt signaling by disrupting β-catenin/BCL9 interaction, are downregulated in tumors; and (iv) disruption of β-catenin/BCL9 interaction^15,16^ is associated with antitumor activity.

The oncogenic activity of BCL9 has only been ascribed to its selective binding to β-catenin, and thus to its role as a Wnt transcriptional co-activator^4,17^. However, induction of Wnt target gene transcription by BCL9 is cell-type-specific and dependent on the cellular context^18^. Moreover, there is growing evidence that BCL9 interacts with proteins other than β-catenin and its oncogenic activity may be, in part, independent of Wnt/β-catenin. For instance: (i) lens development is unaffected in mice with targeted deletion of the BCL9-HD2 domain^19^; (ii) BCL9 acts independently of β-catenin transcription during dental enamel formation^3^; (iii) BCL9 binds to proteins that transmit signals from estrogen and androgen receptors^20^; and (iv) the BCL9/MEF2D fusion protein found in patients with poor-prognosis B-ALL lacks the BCL9-HD2 domain^21,22^. These findings indicate that BCL9 is potentially a multifunctional protein and may have other unrelated roles Wnt/β-catenin co-activation.

In this study, we have identified a novel oncogenic function of BCL9 by using a series of machine learning-based analytical tools. By interacting with paraspeckle proteins, which are involved in post transcriptional regulation, BCL9 stabilizes the mRNA of calcium signaling and neural associated genes to confer neuron-like, multicellular communication properties to a poor prognosis molecular subtype of CRC.

## Results

### BCL9 negatively correlates with survival in a CRC subtype

**Fig. 1:**
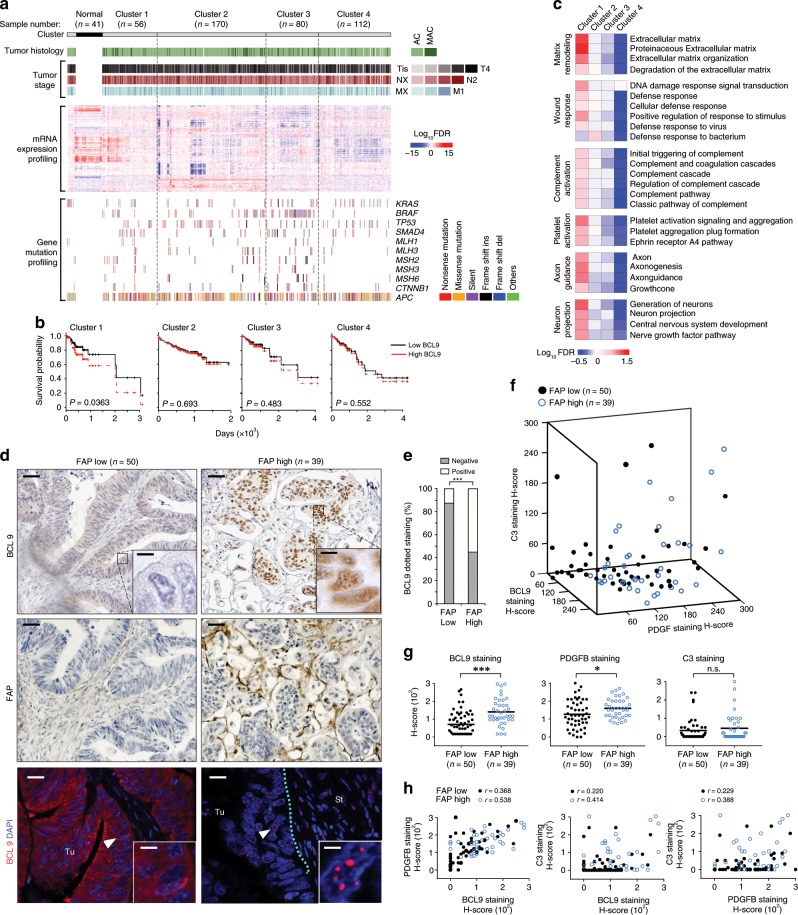
BCL9 expression within specific molecular subtypes of CRC. **a** Top: CRC clusters and the corresponding number, histology and stage of samples. Adenocarcinoma (AC); mucinous adenocarcinoma (MAC); tumor, node, metastasis (TNM). Middle: Heat-map of unsupervised clustering of log2 gene expression levels for CRC clusters. Bottom: type of gene mutation. **b** Cox proportional hazards plot of CRC survival probability according to cluster and BCL9 expression levels (high: top 25%; low: bottom 25%). **c** GSEA of CRC clusters. Color-code response to log10 FDR level (red and blue: high and low confidence, respectively). **d** Representative immunostains of BCL9 and FAP (top) and immunofluorescence (IF) of BCL9 (bottom) according to FAP levels. Note punctate BCL9 nuclear staining in FAP high cases. Tu: tumor; St: stroma. Scale bars: black, 50 µm (top, left) and 10 µm (inset); white, 20 µm (top, left) and 2 µm (inset). **e** Distribution of BCL9 punctate staining according to FAP levels. *P* values were calculated using *χ*^2^ test, ***P < 0.001, **f** 3D scatter plot of immunostain H-scores for the indicated proteins. **g** Immunostain H-scores of indicated proteins in FAP low and high groups. *P* values were calculated using Student’s *t* test, *P < 0.05; ***P < 0.001; ns: not significant. **h** Pearson correlation plots of immunostain H-scores of indicated proteins in FAP low and high groups. See also Supplementary Figs. 1, 2, 3 and Supplementary Data 1.

A cell’s gene expression profile (GEP) reflects its type, and biological behavior^23^. Therefore, we performed unsupervised consensus clustering^24^ of RNA-Seq-based gene expression datasets from TCGA for CRC and normal colon epithelial cells. Among the 418 CRC samples analyzed, up to four distinct molecular clusters (C1–C4) were found (Supplementary Fig. 1a–c), each uniquely characterized by gene expression, but not by histologic, clinical, or gene mutational profiles (Fig. 1a and Supplementary Fig. 1d, e). C1, which comprised 13% of the cases, was the only cluster showing significantly lower survival in samples with high BCL9 expression (Fig. 1b). Detailed analysis of this cluster by gene set enrichment analysis (GSEA) revealed upregulation of gene sets associated with wound healing, tissue remodeling, and neuronal projection such as FAP, PDGFB, C3, and SYP (Fig. 1c, Supplementary Fig. 2a, b and Supplementary Data 1). Estimate analysis of tumor purity revealed that C1 has the most cellular heterogeneity, and tumors within this group were infiltrated by stromal but not by adaptive immune cells (Supplementary Fig. 2c). We validated this observation in another microarray geneset (GSE39582) (Supplementary Fig. 2d, e).

Immunohistochemistry (IHC) of BCL9 and FAP (used as a C1-featured probe and marker of stromal cell infiltration)^25^ on a tissue microarray (TMA) of CRC samples (*n* = 89) revealed that stromal and ganglion cells have the highest level of BCL9 staining in normal colon mucosa (Supplementary Fig. 3a). In tumors however, BCL9 expression was detected in malignant epithelium, and a greater correlation with PDGFB or C3 was observed in high FAP−expressing groups than low FAP groups (Fig. 1d–h and Supplementary Fig. 3b). BCL9 frequently displayed punctate patterns of nuclear staining in the former group (Fig. 1e), which was also observed in a subset of other cancer types and was not correlated with β-catenin staining (Supplementary Fig. 3c–f).

### BCL9 regulates expression of neural-associated genes in C1

**Fig. 2:**
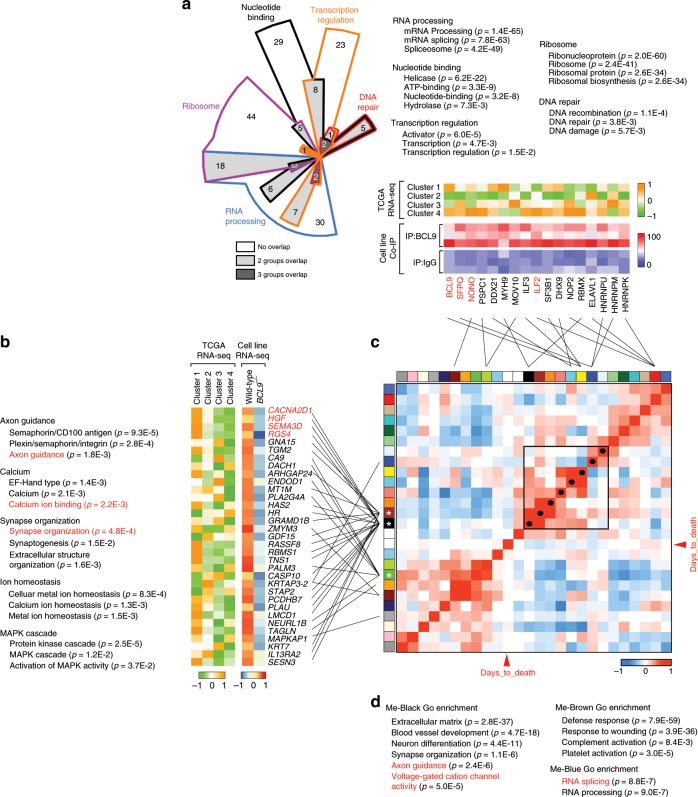
Transcriptional correlation network of BCL9-associated biological processes in CRC subtypes. **a** GSEA Chow-Ruskey diagram of BCL9-interacting proteins as determined by IP coupled with MS analysis. Top: Size of colored areas indicates the number of functional gene sets in each module eigengene (ME). Middle: Heatmap of normalized RNA-seq data of indicated genes and CRC clusters. Colors represent normalized RNA-seq expression levels in CRC clusters. Each row represents the mean value of three independent biological repeats. Bottom: Heatmap of IP coupled MS data. Colors represent total peptide number of indicated proteins. **b** Left: GSEA in pathway gene set for down regulated genes in normalized RNA-seq results of control and BCL9 knockout RKO cells. Middle: heat map of normalized RNA-seq data of indicated genes and CRC clusters. Right: heat map of normalized expression value of RNA-seq results of control and BCL9 knockout RKO cells. **c** Heatmap of the adjacencies in the eigengene network including patient survival for CRC Cluster 1. Outer boxed heatmap: each colored square in the column or row corresponds to one ME or survival time (red arrowhead). Inner boxed heatmap: blue and red colors represent low adjacency (negative correlation), and high adjacency (positive correlation), respectively. Red squares along the diagonal are the meta-modules. Black dots along this diagonal highlight ME displaying a negative correlation with survival time. Colored interconnecting lines indicate group affiliation between ME and BCL9-interacting proteins (top) and genes down regulated in BCL9 knockout RKO cells (left). **d** GSEA of ME-Black (white asterisk), ME-Brown (black asterisk), and ME-Blue (red asterisk) groups. See also Supplementary Figs. 4, 5, 6, 7 and Supplementary Data 2.

We merged gene expression data of 63 CRC cell lines with CRC patients’ samples, and then performed consensus clustering to estimate the similarity of GEP between them and identify representative cellular models of C1 (Supplementary Fig. 4a). The results revealed that Colo320, a well-validated BCL9-dependent cell line^26^ as well as RKO, SW620, and HCT116 cells displayed a GEP similar to the C1 samples (Supplementary Fig. 4b). Representative cell lines for other clusters were also used to perform immunofluorescence (IF). Colo320 and RKO cells displayed the most obvious dotted staining of BCL9 (Supplementary Fig. 4c–e), and showed the greatest decrease in survival among all cell lines analyzed after shBCL9-induced knockdown (Supplementary Fig. 4f, g). Therefore, they were chosen for immunoprecipitation (IP) experiments combined with total protein mass spectrometry (MS) to identify novel BCL9 interacting proteins related to C1, and to better understand the functional consequences of the dotted nuclear staining. Two hundred and fifty proteins were specifically pulled down by anti-BCL9 antibodies(Supplementary Data 2). GSEA revealed these proteins were involved in RNA splicing/processing, transcription regulation, and ribosome function (Fig. 2a). The binding results were further validated by IP using two different anti-BCL9 antibodies, along with MS of protein bands recovered from silver-stained gels (Supplementary Fig. 5a, b), in which VCP, NONO, SFPQ, and ILF2 displayed the greatest number of peptides. In agreement with the size of BCL9 dotted staining in IF (Supplementary Fig 4c), interaction among these proteins was detected in RKO and Colo320 cells, barely so in SW620 and LS174T cells, and not at all in DLD-1 cells by conventional IP (Supplementary Fig. 5c). Colocalization threshold analysis showed that BCL9 co-localizes with NONO, SFPQ, and ILF2 in RKO and Colo320 cells. However, this degree of co-localization is lower compared to NONO and SFPQ in Hela cells, which are regarded as examples of full co-localization^27^, indicating partial BCL9 co-localization with these proteins. (Supplementary Fig. 5d, e).

We performed RNA-seq analysis to identify genes whose expression could be regulated by BCL9 by comparing wild-type vs. BCL9 knockout RKO cells; a list of 975 downregulated genes was selected based on *p*-value (<0.05) and fold-change (>1) (Supplementary Data 3). Levels of NONO, SFPQ, and ILF2 did not change in BCL9 knockout cells (Supplementary Fig. 6a), indicating that expression of these BCL9-interacting proteins is not regulated by BCL9. The top four genes, *RGS4*, *CACNA2D1*, *HGF*, and *SEMA3D* were verified by RT-qPCR (Supplementary Fig. 6b, c). Using GSEA we observed that the genes whose expression was decreased by BCL9 knockout were involved in axon guidance, calcium ion binding, and synapse organization (Fig. 2b, left), and were not enriched as canonical Wnt target genes. Contrary to RKO cells, GSEA revealed that in Colo 320 cells, there was enrichment in canonical Wnt target genes, indicating that BCL9 may play dual functions in this cell line due to the presence of active β-catenin (Supplementary Fig. 6d). Importantly, in PCA analysis, the vector composed of differentially expressed genes between wild-type and BCL9 knockout RKO cells, points towards the C1 direction (Supplementary Fig. 6e). Furthermore, these genes were frequently overexpressed in C1 and its representative cell lines, but not in other CRC patient or cell subtypes (Fig. 2b, right and Supplementary Fig. 6f).

GEP presents a highly ordered structure due to some genes being co-regulated within the same biological processes^28^. We assumed that if BCL9 associated with poor prognosis, then its downstream genes or partners should also be associated with poor prognosis and correlated with each other in the context of C1. Therefore, we employed a global correlation coefficient matrix^29^ to calculate the contribution of each cross-correlated gene set to patient survival (Supplementary Fig. 7a) and to help identify key biological processes driving poor prognosis in C1. When all candidate BCL9-interacting proteins and downstream target genes were projected onto the matrix (Fig. 2c), most of the genes downstream of BCL9, but not the BCL9-interacting proteins, mapped into the Black, Brown, and Blue groups (Supplementary Fig. 7b), which were positively correlated to each other and negatively correlated with survival time (Fig. 2c). Additionally, GSEA revealed that genes in the Black and Brown groups were involved in processes such as extracellular matrix remodeling, neuron differentiation, and wound healing (Fig. 2d). This result was validated in a different TMA (*n* = 84) from the one used in Fig. 1d–g, which showed high levels of BCL9 and RGS4 protein expression in FAP high compared with FAP low groups (Supplementary Fig. 7c); RGS4 displayed a positive correlation with BCL9 expression (Supplementary Fig. 7d, e). These results suggest that BCL9 downstream genes form a co-regulation network that negatively correlates with prognosis, which provide a likely explanation as to why BCL9 expression predicts a poor prognosis in C1 only.

### BCL9 stabilizes the mRNA of neural-associated genes

**Fig. 3:**
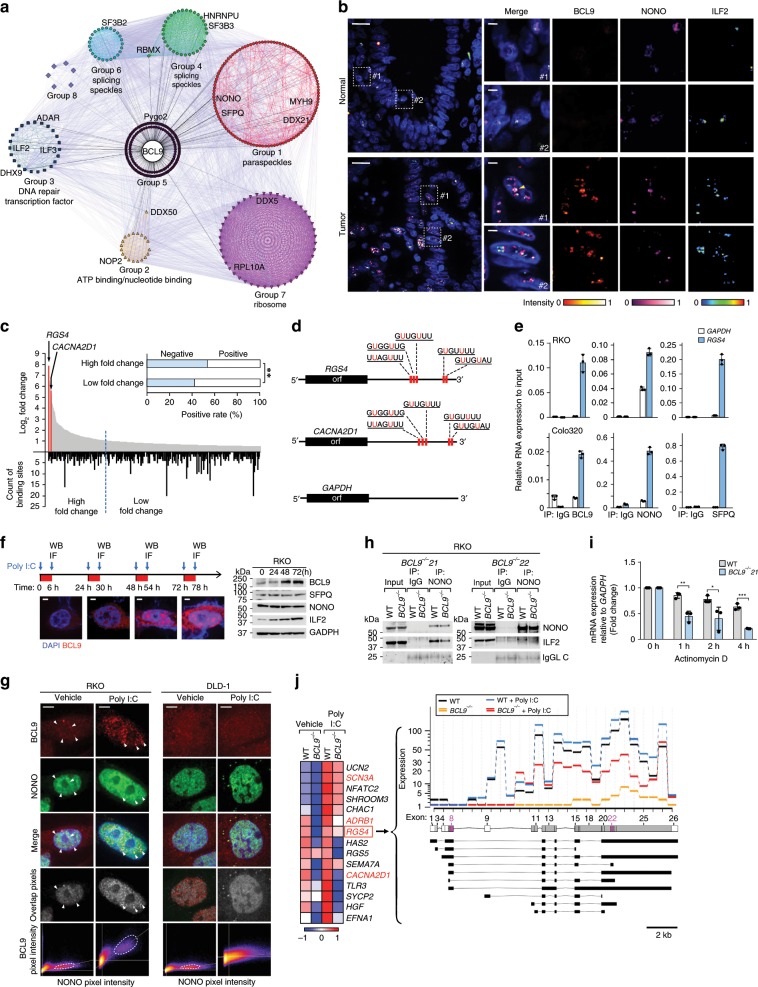
BCL9 regulates mRNA levels of calcium wave-associated genes through paraspeckle proteins. **a** Network of BCL9-interacting proteins identified by Co-IP MS. Each node represents a group of BCL9-interacting proteins with functional relationships. Lines between different nodes represent the “weight” of the protein-protein interaction according to String database (colored) or total peptides in Co-IP assay (black). Groups were clustered by k-means unsupervised classification according to the interacting “weight” among different nodes. **b** Representative IF showing co-localization of BCL9, NONO, and ILF2 in CRC but not in normal colon epithelial cells. Scale bars: 20 µm (top, left), and 2 µm (inset). **c**, **d** SFPQ binding motif in the 3′UTR region of indicated mRNA-encoding genes down-regulated in *BCL9-*knock out RKO cells. High fold change: Genes whose mRNA levels decreased more than 1.5-fold in *BCL9* knockout cells. Low-fold change: others. *P* values were calculated using *χ*^2^ test, **P < 0.01. **e** RIP-PCR v**e**rification of BCL9, NONO, and SFPQ interaction with *RGS4* mRNA in the indicated cell lines. *GAPDH* used as negative control. **f** Time course of BCL9 localization (IF, left) and expression (IB, right) in Poly I:C treated RKO cells. Scale bar: 1 µm. **g** IF co-localization of BCL9 and NONO after 6 h of Poly I:C treatment in the indicated cells. white triangle indicated co-localized pixel of NONO and BCL9. Dotted circle represents a group of pixels which have high intensity of both BCL9 and indicated protein. Scale bar: 2 µm. **h** Co-IP of NONO and ILF2 interaction in two different *BCL9* knockout RKO clones. **i** *RGS4* mRNA expression in actinomycin D treated wild-type and *BCL9* knockout RKO cells. *P* values were calculated using Student’s *t* test, *P < 0.05, **P < 0.01, ***P < 0.001. **j** Left: heatmap of RNA-seq data of wild-type and BCL9 knockout RKO cells treated with vehicle or Poly I:C (*P* < 0.05, *P* values were calculated using Student’s *t* test). Right: highlighted changes of *RGS4* transcript isoforms. Data are displayed as mean ± SD. See also Supplementary Figs. 8, 9, and 10. Source data are provided as a Source Data file.

To further investigate the role of BCL9 in C1, we generated a protein interaction network describing the relationship between all BCL9-interacting proteins identified by Co-IP MS. The proteins were clustered into eight different groups according to the “degree” of their interaction and.functional relationships. We observed a high level of interaction was displayed not only within proteins from the same group, but also from different groups. In support of this interpretation, IF studies revealed that BCL9, NONO, and ILF2, which all belong to different clusters, co-localized in punctate structures around the nucleolus in CRC but not in normal colon epithelial cells (Fig. 3b). IP studies showed that Group 1 and Group 3 proteins co-immunoprecipitated with each other and with BCL9, in Colo320 and RKO cells but not in DLD1 cells (Supplementary Fig. 8a). Notably, Group 1 is enriched in core protein components of paraspeckles. We performed a combination of IF with BCL9 antibody, and fluorescence in situ hybridization (FISH) with non-coding RNA *NEAT1* probe used as a marker of paraspeckles; high intensity BCL9/IF dotted signals were enriched adjacent to and partly co-localized with the *NEAT1*/FISH and NONO/IF signal around the interchromosomal region (Supplementary Fig. 8b, c). We also carried out RNA IP coupled with PCR (RIP-PCR) with anti-BCL9 antibodies and *NEAT1* specific primers in whole cell lysates of RKO cells. As shown in Supplementary Fig. 8d, *NEAT1* was significantly enriched in the anti-BCL9 group. This result, in combination with the previous ISH/IF data, suggests a physical connection and functional link between BCL9 and paraspeckles, but that BCL9 itself is not a core component of paraspeckles. Overexpression of BCL9 in RKO cells increased the viability of wild-type cells but did not rescue or affect the viability of cells with shRNA-induced knockout of NONO or ILF2 (Supplementary Fig. 8e, f) further supporting a functional link. In addition, our observation that BCL9 overexpression did not induce expression of bona fide Wnt downstream target genes in RKO cells (Supplementary Fig. 8g), indicates that in the C1 subtype the effect of BCL9 on cell survival/proliferation depends on its interaction with paraspeckle proteins, but not on the Wnt pathway.

To investigate whether paraspeckle proteins are involved in mRNA processing of BCL9 downstream target genes, we analyzed their 3′UTR regions for the presence of the binding motif for the core paraspeckle protein SFPQ. In support of this hypothesis, the group of mRNAs showing a higher-fold decrease in expression in BCL9-deficient RKO cells were more likely to contain an SFPQ-binding motif than those that showed a low-fold change (Fig. 3c, d). In addition, when RNaseA was added to the protein lysates before adding anti-BCL9 antibodies, the levels of immunoprecipitated SFPQ and NONO, but not of BCL9 and ILF2, were markedly decreased (Supplementary Fig. 9a). Disruption of intranuclear co-localization of BCL9 and NONO after RNaseA treatment was also detected by IF (Supplementary Fig. 9b), indicating that BCL9/NONO/SFPQ but not BCL9/ILF2 interaction is dependent on an intact RNA, and that BCL9 itself should not be regarded as another core paraspeckle protein. Moreover, RIP-PCR of RKO and Colo320 whole cell lysates revealed the presence of RGS4 mRNA within the BCL9/NONO/ILF2 protein complex (Fig. 3e).

We next examined how the BCL9-interacting protein complex regulates expression of target genes downstream of BCL9. When RKO cells were treated with poly I:C, CpG, or LPS to induce formation of paraspeckle complexes^27^, we observed accumulation of BCL9 around the nucleolus after 6 h (Supplementary Fig. 9c). Longer exposure further induced BCL9 accumulation around the nucleolus (Fig. 3f, left), which was also associated with increased BCL9, and ILF2 protein levels (Fig. 3f right and Supplementary Fig. 9d). We observed that: (i) increased levels of BCL9 protein were found in cytoplasmic but not nuclear fractions (Supplementary Fig. 9e), (ii) poly I:C increased the growth of wild-type RKO cells but not *BCL9* knockout RKO cells (Supplementary Fig. 9f), (iii) consistent with our previous results, the location and interaction of BCL9 with paraspeckle proteins in DLD-1 cells was unresponsive to poly I:C (Fig. 3g and Supplementary Fig. 9g, h), and (iv) Poly I:C stimulation increased the partial co-localization between BCL9/IF and *NEAT1*/FISH signals (Supplementary Fig. 9i), and poly I:C reduced the amount of BCL9/β-catenin protein complex (Supplementary Fig. 9j). While Wnt3A increased the interaction between BCL9 and β-catenin, it did not affect BCL9 binding to paraspeckle proteins (Supplementary Fig. 9k). These results indicate that the localization of BCL9 in the nucleoplasm is a dynamic process, and cellular stress, in addition to promoting paraspeckle formation, also increases the interaction of BCL9 with paraspeckle proteins (Supplementary Fig. 9l).

In support of the involvement of BCL9 in RNA splicing/processing, we observed that *BCL9* knockout decreased the interaction between NONO and ILF2 in comparison to wild-type cells (Fig. 3h). In addition, when cells were treated with actinomycin D^30^, the stability of BCL9 downstream target genes was decreased (Fig. 3i). Moreover, RNA-seq studies revealed that compared to untreated wild-type control cells, the BCL9 downstream target genes were increased in poly I:C-treated wild-type RKO cells (Fig. 3j), suggesting a role for BCL9 in processing the mRNA of its downstream targets. In support of this, expression of genes involved in calcium signaling and neural differentiation, such as *RGS4*, *CACNA2D1*, and *ADRB1*^31^, also increased after poly I:C treatment (Fig. 3j). However, in *BCL9* knockout cells, isoforms of *RGS4* mRNA were shorter than in wild-type cells and, even after poly I:C treatment, transcription of the full-length isoform was not rescued (Fig. 3j, right), further supporting a role of BCL9 in mRNA splicing/processing.

We next evaluated whether BCL9 is involved in calcium signaling pathway activation. We treated cells with the adrenergic receptor beta agonist to activate the G protein-coupled receptor-calcium axis^32^. Expression of *RGS4* and *CACNA2D1* mRNAs were increased in both wild-type and BCL9 knockout cells after dopamine treatment; however, in BCL9 knockout cells mRNA expression was not completely rescued to wild-type levels (Supplementary Fig. 10a). In addition, reporter activity dependent on NFATC2, a transcriptional factor whose activity is regulated by calcium waves^33^, was reduced after knocking-down expression of BCL9, ILF2, or CACNA2D1 in RKO and Colo320 cells (Supplementary Fig. 10b–d), suggesting a role for BCL9 in this process via mRNA stabilization of calcium-signaling genes.

### BCL9 regulates communication among CRC cells

**Fig. 4:**
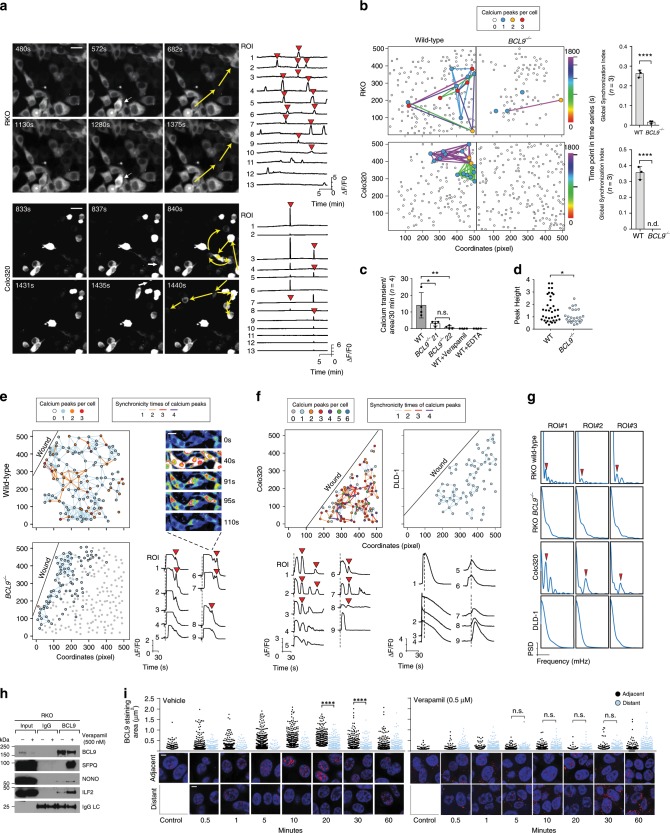
Lack of BCL9 inhibits the occurring and spreading of calcium transients. **a** Time-lapse imaging demonstrating the spread of calcium transients among cells (left), average calcium transient curve (ΔF/F0) of all cells with calcium transients in one microscope field (right). Initiating cell (white arrows), direction of propagation (yellow arrows). Synchronicity of calcium transients is highlighted by red triangle. Scale Bar: 10 µm. **b** Left: network of synchronized calcium transients in the indicated wild-type and *BCL9* knockout cells. Each dot represents one cell, the colored dots indicate the number of calcium wave events during 30 mins. Edged-linked dots represent synchronized calcium transients, colored edge indicates the timing of synchronized calcium transients. Right: synchronicity of global calcium transients in the indicated cells was calculated in wild-type and *BCL9* knockout by FluoroSNNAP, n.d. non-detectable. *P* values were calculated using Student’s *t* test. ****P < 0.0001. **c** Frequency of calcium waves occurring in the indicated wild-type and *BCL9* knockout RKO cells in the absence or presence of Verapamil or EDTA. P values were calculated using Student’s *t* test. *P < 0.05; **P < 0.01; ns: not significant. **d** Peak height of calcium transients in wild-type and *BCL9* knockout RKO cells. *P* values were calculated using Student’s *t* test *P < 0.05. **e** Network of calcium transients spreading (left) and ΔF/F0 of synchronized “secondary waves” (right) after wound scratching in wild-type and *BCL9* knockout RKO cells. Each circle represents one cell, colors represent times of simultaneous calcium transients. Edged-linked dots represent synchronized calcium transients, colored edge indicates times of synchronized calcium transients. Red triangle: “secondary waves”; Black line: wound edge. Scale bar: 5 µm. **f** ΔF/F0 and heatmap showing display of “secondary waves” in Colo320 but not in DLD-1 cells. **g** The frequency spectrum of calcium waves of indicated ROI in parts **e** and **f**. **h** Co-IP of the indicated proteins in RKO cells treated for 2 h with vehicle (−) or verapamil (+). **i** Time-course IF depicting changes in BCL9 staining area in cells located adjacent or distant from scratched edge in vehicle (left) or Verapamil (right) treated RKO cells. *P* values were calculated using Student’s *t* test. ****P < 0.0001; n.s.: not significant. Data are displayed as mean ± SD. Scale bar: 1 µm. See also Supplementary Figs. 11 and 12. Source data are provided as a Source Data file.

We evaluated global calcium changes in CRC cells transduced with GFP-tagged GCAMP5 as a calcium indicator^34^. The occurrence of spontaneous calcium transients was detected in RKO, Colo320, SW620, and LS174T cells which displayed BCL9 dotted staining, but not in DLD-1 cells (Supplementary Fig. 11a, b, and Supplementary Movie 1). As previously shown^35^, we observed filopodia formation after a calcium transient in wild-type but not in BCL9-deficient RKO cells (Supplementary Fig. 11c). Calcium transients were synchronous among individual tumor cells and independent of direct physical contact (Fig. 4a), but lack of BCL9 reduced this synchronicity (Fig. 4b), as well as their amplitude and frequency (Fig. 4c, d). Calcium transients also disappeared after verapamil or EDTA treatments (Fig. 4c). Overall, these results indicate: (i) calcium transient synchronicity is dependent on BCL9, (ii) calcium influx occurs through voltage-dependent calcium gates, and (iii) the source of calcium influx is from the extracellular microenvironment. Because the cells that show synchronicity in calcium influx were not contiguous and did not have direct physical contact, the spreading of calcium influx must be independent of gap junctions^36^ and suggests the existence of a secreted factor. A calcium wave spike library revealed that poly I:C enhanced the length and amplitude of calcium transients in wild-type but not in *BCL9* knockout cells (Supplementary Fig. 11d). Interestingly, knocking down *RGS4* and *CACNA2D1* mRNA levels and treating cells with RGS4 or L-type calcium channels inhibitors phenocopied the effect of BCL9 knockout in RKO cells (Fig. 4a, b vs. Supplementary Fig. 11e–i), further linking BCL9 function with L-type calcium channel associated genes.

It was previously shown in a model of epithelial wound-healing, that one of the first reactions to injury of the cell monolayer is an intracellular calcium rise which spreads as a wave from the injury site to the neighboring cells^37^. By scratching the monolayer cell surface, we elicited calcium transients in the cells closest to the wound edge (hereafter referred to as the “primary” wave) and observed how they spread to distant cells in both wild-type and *BCL9* knockout RKO and Colo320 cells (Supplementary Fig. 12a–d). The amplitude and length of primary waves were significantly reduced and rapidly attenuated during propagation in *BCL9* knockout cells (Supplementary Fig. 12b, d), and only a few distant cells received calcium signaling from the scratched wound edge. Wound healing of the scratched monolayer was delayed in *BCL9* knockout cells (Supplementary Fig. 12a, c). We observed secondary calcium waves in the wound healing assay, which occurred later and were of shorter wavelength than the primary wave (Fig. 4e, f). The timing of “secondary” wave spikes always lagged the primary wave. Although the onset time of the primary wave spike varied among different cells, secondary waves were synchronized, and independent of distance from the wound edge. Frequency spectrum analysis indicated secondary waves were also present in wild-type Colo320 but not in wild-type DLD-1 cells or in RKO and Colo320 cells lacking BCL9 (Fig. 4g).

To further elucidate the functional correlation between BCL9 and calcium signaling, we next investigated whether BCL9 interaction with paraspeckles is responsive to calcium transients. As shown in Supplementary Figs. 12e, f, the display of primary and secondary waves were blocked after verapamil^38^ or EDTA treatment. Co-IP experiments demonstrated the interaction of BCL9 with paraspeckle proteins was increased by verapamil; however, BCL9 protein levels were decreased (Fig. 4h). In addition, a time-course wound healing assay revealed that BCL9 complexation with paraspeckle proteins began ~10 min after the appearance of the primary wave, reaching a peak 20 min later in cells adjacent to the wound in vehicle-treated but not verapamil-treated cells (Fig. 4i). These data indicate that localization of BCL9 complexes with paraspeckle proteins is a response to cellular stress, and that BCL9 might mediate secondary waves via an unknown “extracellular factor”, whose release is dependent upon the opening of voltage-gated calcium channels.

### Neurotransmitters mediate BCL9-dependent TME remodeling

**Fig. 5:**
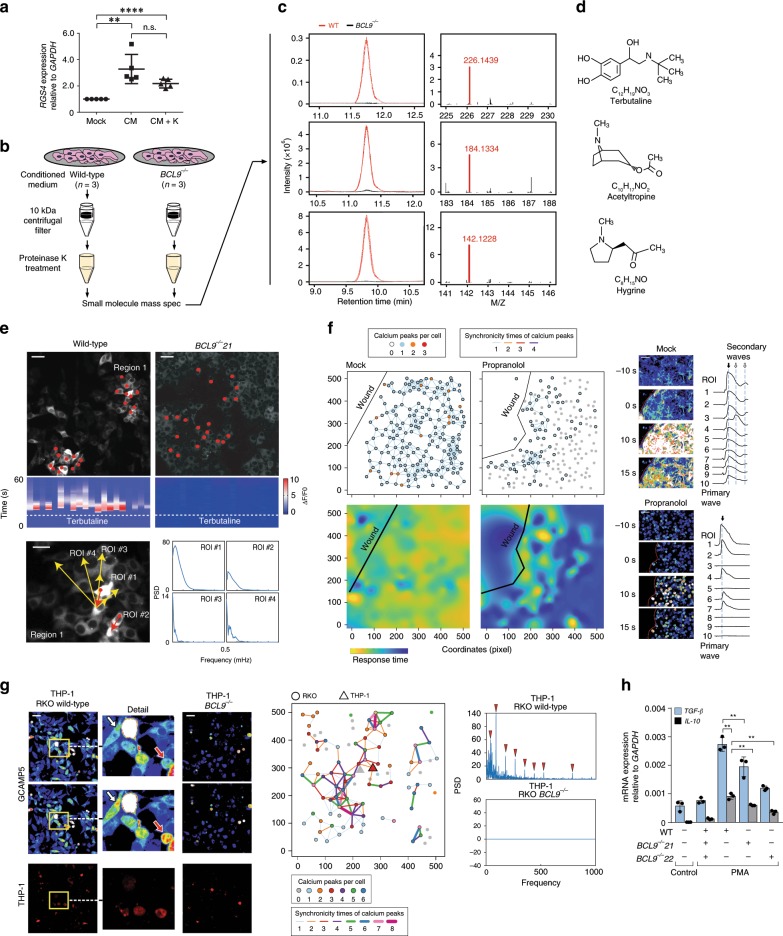
Identification of calcium transient inducers by MS analysis. **a** *RGS4* mRNA expression in five different *BCL9* knockout RKO clones mock treated or treated with conditioned medium (CM) from wild-type RKO cells, with or without proteinase K pre-treatment. *P* values were calculated using Student’s *t* test, **P < 0.01; ****P < 0.0001; n.s., not significant. **b** Diagram of strategy used to identify “extracellular factor(s)” inducers of calcium waves spreading. Retention time and molecular size (M/Z) **c** as well as molecular structure **d** of small-molecules identified by MS in CM from three different *BCL9* knockout RKO cell clones and compared with CM from wild-type RKO cells. **e** Top: heatmap of calcium transient (ΔF/F0) in the indicated ROI (red dots) in wild-type and *BCL9* knockout RKO cells. White dotted line indicates the time of adding Terbutaline to the cell culture medium. Bottom: the frequency spectrum of calcium waves of indicate ROI. Red and yellow arrows arrow indicate the direction of primary and secondary way propagation, respectively. Scale bar: 20 µm. **f** Network of synchronous calcium transients (left, top) of calcium wave spreading and Time-lapse imaging (right) after scratching the monolayer surface in control and propranolol treated RKO cells. Left, bottom: heatmap of cell response time after scratching. Yellow: fast; blue: slow. Scale bar: 20 µm. **g** Left: time-lapse ima**g**ing of calcium waves in THP-1 cells co-cultured with wild-type or *BCL9* knockout RKO cells. White arrows: RKO cells; Red arrows: THP-1 cells. THP-1 cells were transfected with GCAMP5 and dsRed, RKO cells were transfected with GCAMP5 only. Middle: synchronous calcium transient network of wild-type RKO and THP-1 cells co-culture system. Right: the frequency spectrum of calcium waves of wild-type and *BCL9* knockout RKO and THP^-^1 co-culture system. Characteristic spectral lines are marked by red triangle. Scale bar: 20 µm. **h** RT-qPCR analysis of *TGFB2* and *IL10* mRNA expression in THP-1 cells treated with PMA and protein-free CM from wild-type or *BCL9* knockout RKO cells. *P* values were calculated using Student’s *t* test. **P < 0.01. Data are displayed as mean ± SD. See also Supplementary Fig. 13. Source data are provided as a Source Data file.

As shown in Fig. 5a, *RGS4* mRNA expression was increased in *BCL9* knockout RKO cells after treatment with conditioned medium (CM) from wild-type RKO cells regardless of whether CM was pre-treated with proteinase K, suggesting that the extracellular factor is probably not a protein. Protein-free CM from wild-type and *BCL9* knockout RKO cells (Fig. 5b and Supplementary Fig. 13a) revealed the presence of neurotransmitters with chemical structures and molecular weights resembling those of terbutaline, acetyltropine, and hygrine in wild-type but not *BCL9* knockout cells (Fig. 5c, d). This result, taken together with our finding that expression of ADRB1 is increased after poly I:C treatment of wild-type RKO cells (Fig. 3j), suggests that the extracellular factor most likely belongs to the phenethylamine family (i.e. terbutaline). In agreement with this, we observed that the amplitude, frequency, and synchronicity of spontaneous calcium waves was reduced after treatment with propranolol, an inhibitor of ADRB (Supplementary Fig. 13b).

Terbutaline induced propagation of calcium transients in wild-type but not in *BCL9* knockout RKO cells (Fig. 5e, top), however, the occurring time of calcium transients after terbutaline treatment was variable among cells (Fig. 5e, middle) and displayed dissimilar frequency spectrum characteristics (Fig. 5e, bottom). In addition, in the wound healing assay, scratching activated calcium transients in most cells, whilst very few of them were activated in the propranolol treated groups (Fig. 5f, top). In the heat map of response time distribution, cells located at the same distance from the wound edge displayed different response times after scratching (Fig. 5f, bottom). These results indicate that terbutaline was indeed one of the triggers of the calcium transient, and lack of BCL9 reduced the cell sensitivity to terbutaline. Terbutaline treated cells displayed the calcium transient and passed the signal to a specific subset but not to all the cells surrounding them. Therefore, this type of signaling should be regarded as the result of neurotransmitter diffusion and specific functional connections.

This characteristic signaling process resembling neural cells could allow CRC cells to establish a complex communication network on a multicellular scale, and regulate the activity of cells in the TME. To test this possibility, we co-cultured RKO cells with THP-1 cells, the latter being a representative model of human M2 macrophages, which promote tumor progression and TME remodeling^39,40^. Wild-type but not *BCL9* knockout RKO cells transmitted calcium transients to the THP-1 cells (Fig. 5g, left). Synchronous calcium transient network (Fig. 5g, middle) and frequency spectrum (Fig. 5g, right) analysis displayed a complex communication network among these cells in wild-type but not in BCL9-deficient RKO cells, which is an indication of the influence of CRC cells in TME. In support of this view, PMA stimulation (which promotes M2 differentiation) in combination with protein-free CM from wild-type RKO cells, but not from BCL9 knockouts, induced TGF-β and IL10 expression in the THP-1 cells (Fig. 5h). Overall, these results highlight the role of BCL9 in cell-cell communication among CRC cells and with cells from the TME. In keeping with this, we observed that propranolol and verapamil significantly reduced the viability of RKO, Colo320 and SW620 more than LS174T or DLD-1 cells, a finding which could have important implications for CRC therapy (Supplementary Fig. 13c).

### BCL9 is associated with CRC stromal cell infiltration

**Fig. 6:**
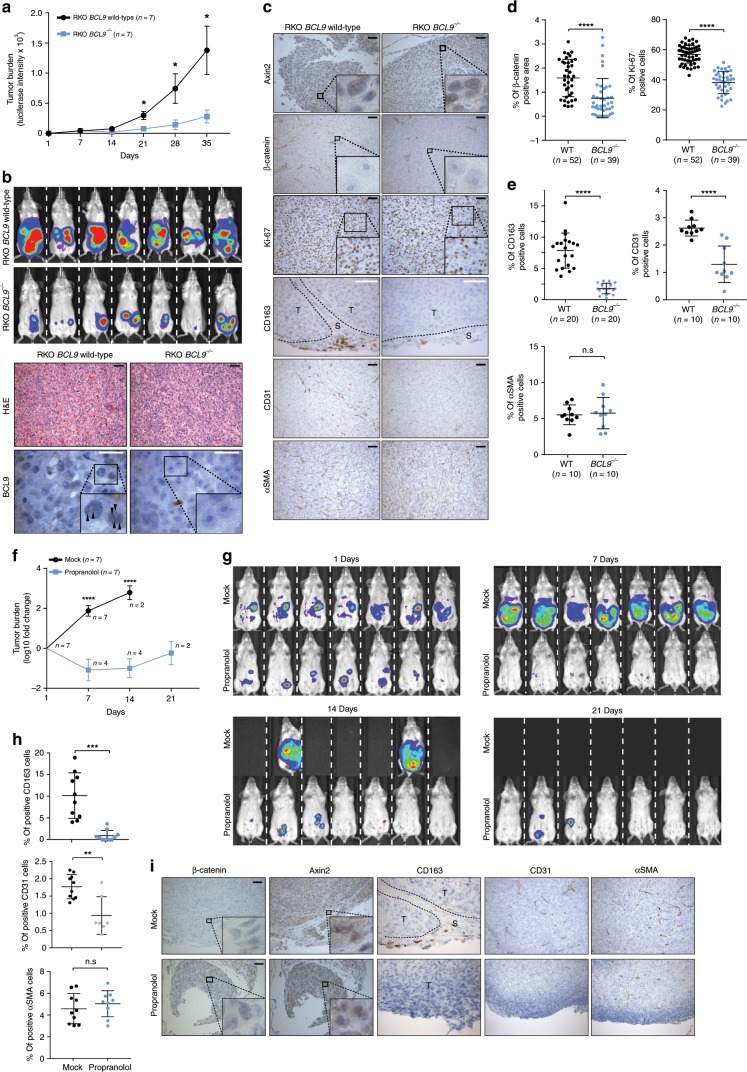
BCL9 knockout, and propranolol treatment reduces CRC progression and stromal cell infiltration in vivo. Top: **a** Tumor burden assessed by body live imaging over time. Each dot represents mean; error bars, standard error of the mean. *P* values were calculated using Student’s *t* test. *P < 0.05. *n* = 7 for both groups. Day 0 indicates first measurement. **b** Top: body live images taken on day 34. Bottom: representative histology and BCL9 immunostain of xenograft tumors. Arrowheads indicates accumulation of BCL9 around paraspeckles. Scale bars: black 50 μm; white, 20 μm. **c** Representative immunohistochemical analysis of xenograft tumors. Scale bars: white, 20 μm; black, 50 μm. **d** Percentage of β-catenin (left) and Ki-67 (right) positive cells per ROI. *N*: number of ROIs analyzed. Tumor nodules dissected from three mice implanted with wild-type or BCL9 knockout RKO cells were used for analysis. Crosses represent mean and standard deviation. *P* values were calculated using Welch’s *t* test. ****P < 0.0001. **e** Percentage of the ar**e**a positive for CD163, CD31, and αSMA staining per region of interest (ROI). *P* values were calculated using Student’s *t* test. ****P < 0.0001. n.s.: not significant. Bottom: **f** Tumor burden assessed by body live imaging over time. Each dot represents mean; error bars, standard error of the mean fold change. *P* values were calculated using Student’s *t* test. *n* = 7 for both groups. ****P < 0.0001. Day 1 indicates first measurement. **g** Body live images taken on days 1, 7, 14, and 21. **h** Percentage of indicated cells of per ROI. *N*: number of ROIs analyzed. *P* values were calculated using Student’s *t* test **P < 0.01. ***P < 0.001. n.s.: not significant. **i** Representative immunohistochemical analysis of xenograft tumors. Scale bars: 50 μm. Data are displayed as mean ± SD.

To investigate the biological consequences of BCL9 interaction with paraspeckles in vivo, luciferase labeled RKO cells, which do not display nuclear β-catenin activity^41^, were implanted intraperitoneally in immunodeficient mice, and tumor growth was evaluated. As shown in Fig. 6a, b (top), tumor growth was significantly reduced in mice implanted with *BCL9* knockout RKO cells. Thirty-five days post-implantation, all mice were euthanized and tumor nodules were harvested and analyzed histologically. Although no major histomorphologic differences were observed between engrafted wild-type and BCL9 knockout RKO cells, the tumor cell component in RKO wild-type engrafted tumors was more heterogeneous than in RKO BCL9 knockout engrafted tumors (Fig. 6b, bottom). As expected, dotted BCL9 staining was detected in wild-type RKO cells but not in BCL9 knockout cells, β-catenin was not detected in any of the RKO cells, and expression of the Wnt target gene Axin2 did not display any differences between the two groups (Fig. 6b, c). Furthermore, Ki-67 immunostains revealed increased numbers of proliferating cells in mice implanted with wild-type compared with BCL9 knockout RKO cells (Fig. 6c, d). We next investigated the cellular component within engrafted tumors by CD163, CD31, and αSMA immunostains, which were used as markers of infiltrating mouse derived M2 macrophages, endothelial and myofibroblast cells, respectively. The number of M2 macrophages and endothelial cells, but not myofibroblasts, were significantly reduced within BCL9 knockout RKO engrafted tumors (Fig. 6c, e). We then investigated the effect of inhibiting calcium influx on tumor burden and infiltration by cells of the tumor TME in mice engrafted with RKO cells, and subsequently treated with vehicle or propranolol in the drinking water. Three weeks after RKO cell injection, all 7 vehicle treated mice but none of the propranolol treated mice had died with extensive tumor burden. Three weeks post tumor injection, except for rare small tumor nodules, tumor cells had disappeared in most of the propranolol treated mice (Fig. 6f, g). Nuclear β-catenin was not detected in RKO cells and expression of Axin2 did not display differences between the control and treated groups. Consistently, propranolol treated mice demonstrated a marked reduction in the number of mouse derived M2 macrophages and endothelial cells, but not of myofibroblast cells, within the TME (Fig. 6h, i). These in vivo results are consistent with our previous in vitro results (Fig. 5g).

## Discussion

Here we have identified a function of BCL9 that is independent of its binding to β-catenin in a C1 poor prognosis molecular subtype of CRC, characterized by high expression of stromal and neural associated genes. Through its interaction with paraspeckle proteins, BCL9 enhances the mRNA stability of neural associated genes and the release of neurotransmitters, therefore sustaining communication among tumor cells and TME remodeling.

**Fig. 7:**
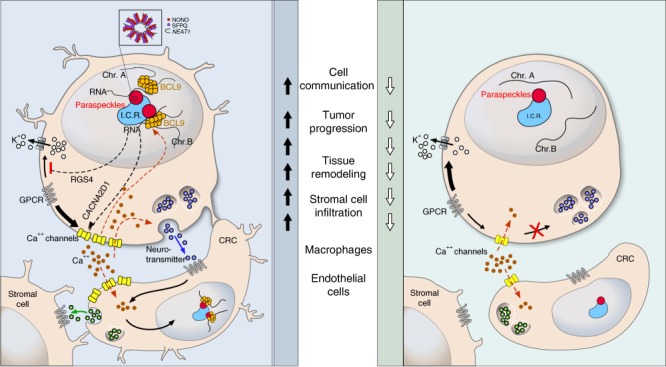
Model for BCL9-dependent upregulation of calcium wave propagation among CRC cells and with the TME. In wild-type C1 CRC cells (left) (e.g. RKO), the generation of spontaneous calcium transients through voltage-gated calcium channel opening, leads to neurotransmitter release and activation of neighboring cells bearing its receptor (e.g. GPCR) to promote calcium release and wave propagation. Simultaneously, CRC cell stimulation and calcium influx promote BCL9 accumulation around and interaction with paraspeckles, generating a positive feedback loop to stabilize mRNA of calcium associated genes (e.g. CACNA2D1) and ensuring the occurrence of the subsequent calcium transients. Thus, BCL9 translocation into paraspeckles provides C1 CRC cells with neuronal-like properties (e.g. cytoplasmic projections, calcium waves) to enhance communication among tumor cells and cells from the tumor microenvironment; this subsequently promotes tumor progression by enhancing tumor progression, tissue remodeling, stromal cell infiltration (e.g. macrophages, and endothelial cells). In C1 cells lacking BCL9 (right), the neuronal-like properties are lost; this is due to the lack of expression of genes associated with the generation of calcium waves, the disruption of the positive feed-back loop, and the fact that calcium waves are not strong enough to induce neurotransmitter release. This consequently terminates the propagation of calcium waves and communication among tumor cells and with the tumor microenvironment. I.R.C. represents interchromosomal region.

Unsupervised clustering of GEP studies has uncovered intertumoral cell heterogeneity and allowed the identification of various molecular subtypes in breast, pancreas, and prostate cancers^42–44^. Similarly, four molecular subtypes of CRC have been previously identified (CMS1-4)^24^. Among them, the CMS4 subgroup is characterized by overexpression of stromal cell infiltration and extracellular remodeling signatures, resembling the C1 cluster identified in our study. This clustering methodology allowed us to identify primary tumor samples and cell lines sharing similar biological behavior, overcoming the problem posed by intertumoral heterogeneity, and eliminating bias from a single biomarker selection^45^. Using the same clustering approach and the presence of dotted nuclear staining of BCL9, we identified C1 cell lines for in vivo functional studies. These studies are consistent with a model in which BCL9 promotes neural-like behavior of C1 cells, including the induction of propagating calcium transients and secretion of neurotransmitters. By enhancing communication among tumor cells and cells from the TME, BCL9 promotes tumor progression by increasing tumor growth, tissue remodeling, and infiltration by stromal cells (Fig. 7).

In this model, Toll-like receptor-induced cellular stress promotes calcium overload, enhancing accumulation of BCL9 adjacent to paraspeckles. After binding to paraspeckle proteins by a currently undetermined mechanism, BCL9 stabilizes the mRNAs of neuronal-associated functional genes. Consistent with our model, RNA-seq, live cell imaging and small molecular MS analyses revealed that lack of BCL9 decreased the expression of voltage-dependent calcium channel and synapse-organizing associated genes, which inhibited calcium transients and neurotransmitter release. Among the mRNAs stabilized by BCL9 was *RGS4*, a regulator of alpha units of heterotrimeric G proteins^46^ which is broadly expressed in excitable tissues such as brain cortex^47^ and smooth muscle^48^. Other mRNAs stabilized by BCL9 include the calcium channel encoding genes *CACNA2D1*; they are also highly expressed in neuronal cells and their opening is triggered by cell membrane depolarization and G-protein signaling activation^49^. The role of BCL9 in neuronal cells was supported by high BCL9 expression in ganglion cells but not in normal colon epithelial cells. In addition, an association between abnormal expression of BCL9 in the brain cortex and negative symptoms in patients with schizophrenia, which is attributed to abnormal activation of calcium signaling and dopamine secretion^50^ has been observed previously^51,52^. Therefore, as postulated in other tumors^53^, it seems feasible that C1 CRC cells have hijacked BCL9 function to resemble neuronal cells, allowing them to communicate.

Paraspeckles are subnuclear bodies localized within the interchromatin space, adjacent to nuclear speckles, and play a critical role in the control of gene expression during many cellular processes including differentiation and stress response^54^. Paraspeckle formation is a dynamic process involving the recruitment of DBHS proteins (NONO and SFPQ) and *NEAT1* from the nucleoplasm, to the gene locus that is undergoing transcription^55^. Similarly, we show that multiple copies of BCL9 are recruited adjacent to paraspeckles from a pre-existing pool in the nucleoplasm through a dynamic process that is independent of Wnt activity. Importantly, BCL9 is not needed for paraspeckle formation. Contrary to the formation of paraspeckles, the interaction between BCL9 and paraspeckle proteins is CRC cell type specific. This seems to explain why expression levels of *NEAT1* do not show differences among different CRC clusters. Similar to BCL9, other non-DBHS proteins including BCL6^56^ and SOX9^57^ transcriptional factors have also been shown to interact with paraspeckle proteins. In addition to cell stress, cell membrane depolarization also induces paraspeckle formation^58,59^. Accordingly, we show that accumulation of BCL9 near paraspeckles occurs after calcium influx. This process could be considered a positive regulatory mechanism, which ensures that the calcium signaling associated system works normally during cellular stress. By interacting with paraspeckle proteins, BCL9 regulates the spontaneity of calcium transients, and enhances communication between C1 cells but not from other CRC clusters.

We identified spontaneous calcium transients among C1 CRC cells, in which spreading was dependent on specific projection of neurotransmitters and the induction of calcium channels opening in distant cells. Similar phenomena have also been described in other cancer types^60,61^, including breast cancer, in which calcium influx was found to be driven by the TRP or ORAI family of calcium channels^62,63^. Expression of these calcium channels is also increased in C1 compared with other CRC clusters, and like L-type calcium channel genes (e.g. CACNA2D1), many of the TRP or ORAI genes also belong to the “black” group in the correlation coefficient matrix, indicating a synergy between these proteins in C1 tumors. In wound healing assays, the spread of calcium transients in *BCL9* knockout cells was blocked in most of the cells adjacent to the wound edge. In contrast, propranolol treatment inhibited calcium transients only in a subset of cells, indicating that several types of neurotransmitter might be involved in the spread of calcium transients in C1 BCL9 wild-type cells. The identification of several molecular structures including terbutaline-like compounds by MS analysis is consistent with this scenario. Spreading of calcium transients among C1 cells is not promiscuous and follows a cell to cell specific pattern, allowing CRC cells to establish a complex communication network. This communication system can reduce the response time to stimulation and rapidly transmit information to distant cells. Like normal neural cells, which have been shown to regulate the behavior of M2 resident macrophages in normal colon mucosa and participate in the formation of a neural-immune network^64^, C1 cells regulated M2 macrophages differentiation in vitro and tumor infiltration in vivo. This is reflected by the strong correlation of the innate immune and neural gene signatures in the correlation coefficient matrix.

In summary, we found that BCL9 promotes neural features through its interaction with paraspeckle proteins in a specific subtype of CRC, and enhances tumor progression by promoting neurotransmitter-dependent communication between tumor cells and cells of the TME. Therefore, this work provides additional insights into the role of BCL9 in tumor progression, and points towards new avenues for therapeutic intervention by targeting BCL9 itself or blockade of neurotransmitter receptors or calcium channels with FDA approved drugs^65,66^.

## Methods

### Ethics statements

We have complied with all relevant ethical regulations for animal testing and research. All animal experiments were approved by the Institutional Animal Care and Use Committee of the Dana-Farber Cancer Institute. Usage of human material was approved by the Dana-Farber Cancer Institute Institutional Review Board, in compliance with the Helsinki Declaration.

### Generation of *BCL9* knockout cell lines by CRISPR-Cas9 system

To generate a *BCL9* knockout cell line, lentiCrisprV2 plasmid (#52961) containing gRNA targeting either *BCL9* (CAGTAGTTTGGCCATGGGA) or *AAVS1*^67^ was delivered to RKO or Colo320 cells using lipofectamine 2000 (Life technologies). After 48 h transfection, cells were cultured in 96-well plates according to a serial dilution protocol (Cell Cloning by Serial Dilution in 96-well Plates, Corning Incorporated Life Sciences). Single clones from the 96-well plates were harvested once the cell counts reached 1 × 10^2^ and were transferred to 12-well plates to continue growing. The expression level of BCL9 was analyzed by IB. Genomic DNA was extracted, the gRNA targeting sequencing was amplified by PCR, and the PCR product was sequenced to identify the frameshift mutation of *BCL9*. To limit off-target effects caused by continued expression of gRNA and Cas9, we did not use puromycin selection. The gRNA was specifically designed to target the 5’UTR portion of the BCL9 ORF which codes for the amino acid sequence between the HD1 and HD2 domain; this creates a frameshift mutation which induces loss-of-function of BCL9 but simultaneously preserves the HD1 domain. Mutated BCL9 is therefore still able to occupy the Pygo2 binding site, which further eliminates the possibility that other co-factors will compensate for BCL9 function.

### Gene expression profiling and statistical analysis

RNA was extracted from wild type and BCL9 knockout RKO cells with or without Poly I:C treatment (1 μg/ml) and subsequently purified using the TURBO DNA-free™ Kit (AM1907, invitrogen) to remove any residual DNA. An RNA library was prepared using the ribosome RNA removing method and sequenced with a 150-bp paired-end protocol in the Center for Cancer Computational Biology at the Dana Farber Cancer Institute (GSE140488). After quality control (QC) analysis was performed using fastQC, the first 10 bases were trimmed for each read. STAR software was used to map the readings to the human genome (hg19) and duplicates were removed using Picard. HTSeq was used to evaluate the gene expression levels by the count number for each gene and were subsequently annotated using the Ensemble database. Gene differential analysis was then applied to the expression profiling table using R package edgeR. For further analysis, the difference in exon usage between different conditions was calculated and R package DEXSeq was used to find differences between them.

### Coimmunoprecipitation assays

To extract nuclear protein, cells were incubated with cytoplasmic lysis buffer (50 mM Tris-HCl pH = 8.0, 20 mM NaCl, 2 mM EDTA, 0.5% Tween-20 containing protease/phosphatase Inhibitor, #5872, Cell signaling) on ice for 10 mins, centrifuged at 6000 × *g*, and the precipitate collected. This step was repeated three times to remove at much cytoplasmic protein as possible. Subsequently, nuclear lysis buffer (50 mM Tris-HCl pH = 7.4, 150 mM NaCl, 2 mM EDTA, 1% Triton X-100(v/v) containing protease/phosphatase inhibitor) was added to the precipitate, and sonication was used to lyse the sample before centrifugation at 16,000 × *g* for 15 min at 4°. In all, 1 mg of the nuclear lysate was blocked with 5% BSA for 1 h at 4°, before overnight incubation with anti-BCL9 (1:500 dilution, ab37305 Abcam, 6109 generated in New England Biolabs), NONO (1:500 dilution, ab70335, Abcam), SFPQ (1:500 dilution, ab38148), ILF2 (1:500 dilution, H00003608-D01, Abnova), or β-catenin (1:500 dilution, 9562L, Cell signaling) antibodies. Normal rabbit IgG (1:250 dilution, sc-3888, Santa Cruz) was used as a negative control. The following day, Protein G and A DynaBeads (10003D,10002D, ThermoFisher) were added to the nuclear lysate at a ratio of 1:1 and rotated for an additional 4 h. The beads were washed three times with washing buffer (50 mM Tris-HCl pH = 7.4, 200 mM NaCl, 2 mM EDTA, 1% Triton X-100(v/v)). The beads were then re-suspended with 2X LDS sample buffer and boiled for 10 mins. For Immunoblotting, the sample was electrophoresed using SDS-PAGE, transferred to nitrocellulose membrane and blocked using non-fat 5% milk. The membrane was subsequently probed using anti-BCL9 (1:1000 dilution, H00000607-M01, Abnova), NONO (1:1000 dilution, TA504777, Origene), SFPQ (1:1000 dilution, MA1-25325, ThermoFisher), ILF2 (1:5000 dilution, PA5-18718, ThermoFisher), or β-catenin (1:1000 dilution, 610154, BD Transduction Laboratories) antibodies. For total protein MS analysis, IP protein samples from anti-IgG and anti-BCL9 groups were recovered by Trichloroacetic acid (TCA 47658-U, Sigma) precipitation; samples were incubated for 10 min at 4°, before centrifugation at 16,000 × *g* for 5 min. In addition, pulled down protein samples were also analyzed by silver staining; bands which existed in anti-BCL9 samples, but not in the anti-normal IgG groups, were cut and used for further MS analysis to validate our previous results. All mass spectrometry was performed in the Taplin Mass Spectrometry Facility at Harvard Medical School (PXD016172) [10.6019/PXD016172]. To limit any off-target effects of anti-BCL9 antibody, we verified the MS results with two independent anti-BCL9 antibodies (37305 Abcam and 6109) which target different amino acid sequences of BCL9. In RNA Immunoprecipitation (RIP)-PCR assay, pull-down RNA was extracted from DynaBeads by using Trizol solution and subsequently analyzed by qRT-PCR.

### Consensus clustering analysis

This study used RNA sequencing data from CRC patient samples from TCGA (https://cancergenome.nih.gov/) and GSE39582 as well as CRC cell lines dataset from these two sources^68,69^. Unsupervised clustering was performed using R Package Consensus Cluster Plus. Gene expression data was normalized by the data size factor of the R package DESeq. The top 2000 genes with the biggest variation in expression were used to generate sample clusters. According to the efficiency of the different number of clusters (K), the patient samples were clustered into 4 groups. The heat map demonstrates the expression of all genes in the 4 clusters. Cox proportional hazards survival analysis was then used to determine whether there is a correlation between survival and the expression level of BCL9 in different clusters. To investigate differential gene expression in each of the 4 clusters, the ANOVA statistical test (followed by post hoc testing) was used, and a *P* value of <0.01 was considered significant. After identification of the gene expression sets, the enrichment score of each gene was calculated using MSigDB C2 pathway gene sets. GO analysis was applied to summarize the function of specific genes by using SP_PIR_KEYWORDS annotation categories in DAVID. The genes that appeared in both the BCL9 correlated gene set and cluster specific gene set were used to calculate the enrichment score by MSigDB C2 pathway gene sets. The correlation coefficient network was generated with gene expression data from C1 tumor samples using the R package WGCNA. Patients in the same cluster shared a similar gene expression profile but displayed different survival times; we observed that those patients with a shorter survival time were sampled at a later stage of the disease, therefore survival time was used to evaluate tumor progression. RNA-seq and Mass spec analysis were carried out independently from each other. The purpose of this was to describe the synergistic effect of BCL9 downstream genes or BCL9 partners, and to ensure the BCL9-regulated bio-events existed and were observed during tumor progression.

### Protein interaction network and GO analysis

The differential nuclear location of BCL9 implied it may interact with multiple types of protein complex therefore we established the protein interaction network to evaluate the intensity of these interactions. To normalize the samples, any proteins in which the peptide number in anti-lgG was higher than the matched anti-BCL9 group (i.e. the protein did not exist in at least two independent experiments) were removed. The enrichment score was calculated by subtracting the total lgG peptide value from anti-BCL9. After normalization, 276 proteins demonstrated a score above 2 and were chosen for further analysis. GO analysis in DAVID was used to define the functional groups of the proteins, and the result was presented in Chow-Ruskey diagrams. The candidate proteins were used to generate the protein-protein interacting network; in the map, proteins are represented by a colored dot and the interactions among individual proteins are represented by connected colored lines. Their weight corresponds to the combined score which was collected from the String database (String database). Then, using the weight score for all the protein interactions as the distance between two proteins, and using a K-mean algorithm to cluster them, the proteins were found to cluster into 7 groups. The number of groups was determined by the first K-value > 2 which has a near stable SS (Sum of squared error). The number of SFPQ binding motif in 3′UTR region of BCL9 downstream genes are identified by using RBP map website.

### Cell viability assays

All cell lines used in this study (RKO, Colo320, SW620, HCT116, LS174T, HT29, SW403, DLD-1, HCT15, SW48, and THP-1) were from ATCC, authenticated by the short tandem repeat (STR) DNA method at the Human Cell line Identity Verification facility at Dana-Farber Cancer Institute or using in our own lab the *Promega Power Plex 16HS Kit*, and tested for mycoplasma contamination using e-Myco, a Mycoplasma PCR Detection Kit (Abbott). Cells that were transfected with shRNA and had undergone puromycin selection were plated into 96-well plates (in triplicate) at 4 × 10^3^ cells/well. The cell Titer glow viability assay (Promega G7570) was used according to the manufacturer’s instructions; briefly the cells were cultured for 48 h, incubated with cell titer glow substrate and analyzed using a luciferase reader. The background luminescence of a blank well was subtracted from the sample readings.

### Wound healing assays

Wild-type or *BCL9* knockout cells were grown in 6-well plates or Nunc™ Glass Bottom Dishes (150680, ThermoFisher) for 24 h until cells reached 90% confluency. A 1-ml pipette tip was used to scratch the monolayer of cells across the center of each well. The cells were imaged at 0 and 24 h after scratching. To investigate the effect of BCL9 on calcium waves (as seen in Fig. 4i), cells were cultured in glass-bottomed 96-well plates for 24 h until they reached 90% confluency. Verapamil (500 nM, V4629-1G Sigma) or DMSO was added the cell culture medium 1 h before scratching. A 200-µl pipette tip was used to scratch the cell monolayer from the top left to the bottom right corner of the well. At various times after the scratch was made, cells were fixed with 4% paraformaldehyde for 15 min at room temperature. IF was subsequently used to identify the location of BCL9 (see IF method section for more detail). We defined the wound edge in the center of the well as “adjacent” and the area in the top right corner was defined as “distance”.

### In vivo mouse xenograft model

A total of 1 or 4 × 10^6^ wild-type or BCL9 knockout RKO cells stably transduced with a reporter expressing Luciferase were injected intraperitoneally into CB17.Cg-PrkdcscidLystbg-J/Crl (Beige) mice (*n* = 7 per group), and tumor burden was monitored by whole-body imaging using Xenogen system every week for 4 weeks, starting one day after injection of cells. After last imaging, all mice were euthanized, and all tumor nodules identified in the peritoneal cavity were dissected and processed for histological and immunohistochemical analysis. To quantify β-catenin and Ki-67 stains serial consecutive sections were scanned using Vectra 2 Intelligent Slide Analysis system; percentage of positive cells (Ki-67, CD163, CD31, or αSMA) or areas (β-catenin) were assessed using inform Cell Analysis software (PerkinElmer). *P*-values were calculated using unpaired Student’s *t*-test. Propranolol hydrochloride (P0884 sigma) was added to the drinking water at a concentration of 0.5 g L^−1^ on the injection day. Drug solution was renewed every 2 days.

### Immunohistochemistry

TMA sections (from Oncology Pathology, Dana Farber Cancer Institute) were pre-heated at 65° for 20 mins and then deparaffined by performing the following washing steps: xylene 3 × 5 mins, 100% ethanol 5 mins, 95% ethanol 5 mins, 75% ethanol 5 mins, 50% ethanol 5 mins, 25% ethanol 5 mins, and rinsed by cold water. Sections were subsequently heated in a microwave with antigen retrieval buffer (10 mM Tris Base, 1 mM EDTA Solution, 0.05% Tween 20, pH 9.0). Sections were washed twice with TBS plus 0.5% Triton X-100 (v/v) and blocked with 5% BSA for 1 h at room temperature. Sections were incubated overnight with the following antibodies at 4 degrees: BCL9 (1:500 dilution, ab37305, Abcam), FAP (1:200 dilution, AF3715, R&D), β-catenin (1:500 dilution, 610154, BD Transduction Laboratories), SYP (1:200 dilution, PA0299, Lecia), PDGFB (1:200 dilution, ab23914, Abcam), C3 (1:200 dilution, HPA003563, sigma), RGS4 (1:200 dilution, sc-398348, Santa Cruz), CD163 (1:250 dilution, 182422, Abcam,), CD31 (1:250 dilution, 77699, Cell signaling), or aSMA (1:250 dilution, 19245, Cell Signaling), Axin2 (1:250 dilution, #2151, cell signaling). The sections were washed three times before incubation with secondary antibody for 2 h at room temperature. The sections were then washed three times before signal amplification with HRP polymer or fluorescence. ImageJ2 software was used to carry out semi-quantitative analysis of the staining intensities, which ranged from 0 (negative) to 3 (strong staining). Subsequently the H-score was calculated using the following formula;

1 $${\mathrm{H}} - {\mathrm{Score}} = \left( {{{\% }}\,{\mathrm{at}}\,{{0}}} \right) \times {{0}} + \left( {{{\% }}\,{\mathrm{at}}\,{{1}} + } \right) \times {{1}} + \left( {{{\% }}\,{\mathrm{at}}\,{{2}} + } \right) \times {{2}} + \left( {{{\% }}\,{\mathrm{at}}\,{{3}} + } \right) \times {{3}}$$

The intensity of staining and cell numbers were detected by imageJ2. The top 50% score of FAP was identified as “FAP high”, and the lower 50% score was identified as “FAP low”.

### Immunofluorescence and fluorescence in situ hybridization

Cells were cultured on corning Glass Coverslips (354085, BioCoat) contained within the wells of a 24-well plate for 24 h, and then fixed using 4% paraformaldehyde. Cells were washed three times with PBS, permeabilized with 0.5% Triton X-100 (Tris-HCl pH = 7.6, 150 mM NaCl, 0.5% Triton X-100 (v/v), washing buffer) for 15 min at room temperature, and blocked with 5% BSA for 1 h at room temperature. The cells were incubated overnight at 4° with the following antibodies; anti-BCL9 (1:500 dilution, ab37305, Abcam), NONO (1:100 dilution, TA504777, Origene), ILF2 (1:500 dilution, PA5-18718, ThermoFisher) or β-catenin (1:500 dilution, 610154, BD Transduction Laboratories). The following day, cells were washed three times with PBS and incubated with fluorescently tagged anti-mouse (1:1000 dilution, A11029, ThermoFisher), rabbit (1:1000 dilution, A11035, ThermoFisher), or goat (1:1000 dilution, A21447, ThermoFisher) secondary antibodies for 1 h at room temperature. Cells were washed three times with PBS and stained with DAPI (D9542, Sigma) for 2 mins. Cells were subsequently washed three times with PBS to remove any residual DAPI stain, and then imaged with an SD confocal microscope. The colocalization threshold is calculated by ImageJ2. Dotted staining area of BCL9 was calculated by the following step: confocal images were processed by High Frequency Signaling Removal to filter out the rapidly changing signal. This process removes the noise and non-dotted staining of BCL9. Then, the top 2% signaling areas were selected. High Frequency Signaling Removal and area size was calculated by ImageJ2. *NEAT1* and fluorescence probe was purchased from Biosearch Technology (*NEAT1* SMF-2036-1). IF combined FISH of BCL9/*NEAT1* was performed as recommended by the supplier.

### Quantitative real-time polymerase chain reaction

RNA was extracted from cells or DynaBeads by using Trizol solution and converted to complementary DNA using a High Capacity cDNA Reverse Transcription Kit (4368814, ThermoFisher). Quantitative PCR was carried out using SYBR Green Master Mix (4309155, ThermoFisher) and the results were normalized to GAPDH expression. Please see Supplementary Table 1 for a list of PCR primers that were used in this study.

### Immunoblotting

Cells were lysed using RIPA Buffer (50 mM Tris-HCl pH 7.5, 150 mM NaCl, 1 mM EDTA, 1% NP-40, 0.1% SDS, 0.5% sodium deoxycholate) with addition of protease and phosphatase inhibitor (#5872, Cell signaling). Following lysis, cells were incubated on ice for 10 min and centrifuged for 10 min at 4° at 16,000 × *g*. In all, 40 μg of each protein lysate was electrophoresed using SDS-PAGE, transferred to nitrocellulose membrane, blocked with 5% non-fat milk, and incubated overnight with following antibodies; anti-BCL9 (1:1000 dilution, ab37305, Abcam), NONO (1:1000 dilution, TA504777, Origene), SFPQ (1:1000 dilution, MA1-25325, ThermoFisher), ILF2 (1:5000 dilution, PA5-18718, ThermoFisher), TLR3 (1:500 dilution, ab62566, Abcam), β-catenin (1:1000 dilution, 610154, BD Transduction Laboratories), CD44(1:1000 dilution, #3570, Cell signaling), Flag tag (1:1000 dilution, A8592, sigma), Axin2 (1:1000 dilution, #2151, Cell signaling), p65 (1:1000 dilution, #8242, cell signaling), LaminB1 (1:1000 dilution, sc-6216, Santa Cruz), and GAPDH (1:1000 dilution, ab9485, Abcam). Secondary antibodies conjugated to horseradish peroxidase were purchased from Santa Cruz Biotechnology (1:5000 dilution, sc2020, Santa Cruz) and Cell signaling (1:5000 dilution, #7074, #7076, Cell signaling).

### Reporter assays

Cells were co-transfected with shLacZ, shBCL9, shILF2 plasmids, and subsequently transfected with NFAT luciferase reporter (Plasmid #10959, Addgene) and SV40 drive renilla luciferase reporter (E2231, Promega). 24 h post-transfection, 1 × 10^5^ cells were plated per well in 96-well plates. 24 h after seeding, the Dual-Luciferase Reporter Assay System (Promega) was used to measure luminescence according to the manufacturer’s instructions. The luciferase signal from the NFAT reporter was normalized to the luciferase signal from the renilla reporter.

### shRNA, siRNA, and ORF expression

The shNONO-1, shNONO-2, shILF2-1, and shILF2-2 plasmids were purchased from Sigma. shBCL9, shLacZ and the BCL9 expression ORF were obtained from Ruben Carrasco’s lab, and the shRNA sequences are noted in Key resource table. A plasmid was used to express BCL9 ORF. *RGS4* and *CACNA2D1* siRNAs were purchased from Sigma (siRGS4#1 SAS1_Hs02_00323157, siRGS4#2 SAS1_Hs02_00323155, siCACNA2D1#1 SAS1_Hs02_00303191, siCACNA2D1#2 SAS1_Hs02_00303142). Lentivirus was produced in 293T cells using the three-vector system. Virus was diluted 1:1 to culture medium and added to cells in a T25 flask containing 1:1000 (v/v) polybrene (sc134220, Santa Cruz). 10 µg ml^−1^ puromycin was used after 48 h infection to screen infected cells. siRNA was transfected by lipofectamine 2000 (ThermoFisher 11668030).

### Small molecular Mass spec analysis

Cells were cultured in 6-well plates for 24 h in antibiotic free medium with 10% FBS. The cell culture medium was collected and purified using a 10kd filter (MRCPRT010, Microcon-10kDa Centrifugal Filter Unit). In addition, the medium was incubated with proteinase K (P6556, sigma) for 1 h at 37°. Samples were sent for MS/MS Mass spec analysis at the Small Molecule Mass Spectrometry Facility, Harvard University.

### Quantification and analysis of calcium imaging data

To trace the calcium transient in real time, GCaMP5G (#31788, Addgene) was transfected into wild-type or *BCL9* knockout RKO and Colo320 cell lines. In all, 2 mg ml^−1^ neomycin was added to the cell culture medium 48 h after transfection to generate cell lines with stable GCaMP5 expression and removed one week prior to the initiation of experiment. Calcium transients were captured by live-cell imaging using a confocal microscope. Time lapse imaging was setup so that an image was captured every 0.5 s for 30 min, with an exposure time of 0.5 s. Calcium measurements were normalized to background fluorescence and the relative change in calcium (*F(t)*) over time was calculated by the formula:

2 $$F\left( t \right) = \frac{{F - F_0}}{{F_0}} = \frac{{\Delta F}}{{F_0}}$$

where *F*_0_ was defined as the average intensity of the 20% lowest gray values in a region of interest (ROI), *F* was defined as the normalized gray values of ROI in time point *t*. Global synchronicity was calculated by cross correlation method in FluoroSNNAP. The frequency spectrum was calculated by fast Fourier transform^70^, the time difference between each sample is 0.5 s.

### Statistical analysis

Statistical significance was evaluated in GraphPad Prism using the unpaired Student’s *t*-test. A *P*-value ≤ 0.05 was considered statistically significant.

### Reporting summary

Further information on research design is available in the Nature Research Reporting Summary linked to this article.

## Supplementary information

Supplementary Information

Description of Additional Supplementary Files

Supplementary Data 1

Supplementary Data 2

Supplementary Data 3

Supplementary Movie 1

Reporting Summary

## Data Availability

The RNA sequencing data has been deposited in the NCBI GEO database (GSE140488) and the proteomic data has been deposited in the ProteomeXchange database (PXD016172) [10.6019/PXD016172]. The source data underlying Figs. 3e–f, 3h–I, 4h, and 5a, h, and Supplementary Figs. 4f–g, 5a, c, 6a–c, 8a, d–g, 9a, e–h, j–k, 10a–d, 11e, and 13c are provided as a source data file.

## Source data

Source Data
