# Supplementary material for: BCL9 provides multi-cellular communication properties in colorectal cancer by interacting with paraspeckle proteins
Source: Nat Commun. 2020 Jan 7;11:19. doi: 10.1038/s41467-019-13842-7 (PMC6946813; doi:10.1038/s41467-019-13842-7)
Supplement: Supplementary file 1 — Supplementary Information [file 41467_2019_13842_MOESM1_ESM.pdf]

Supplementary Information

BCL9 provides multi-cellular communication properties in colorectal cancer by interacting with paraspeckle proteins

Jiang *et al.*

Supplementary Figure 1

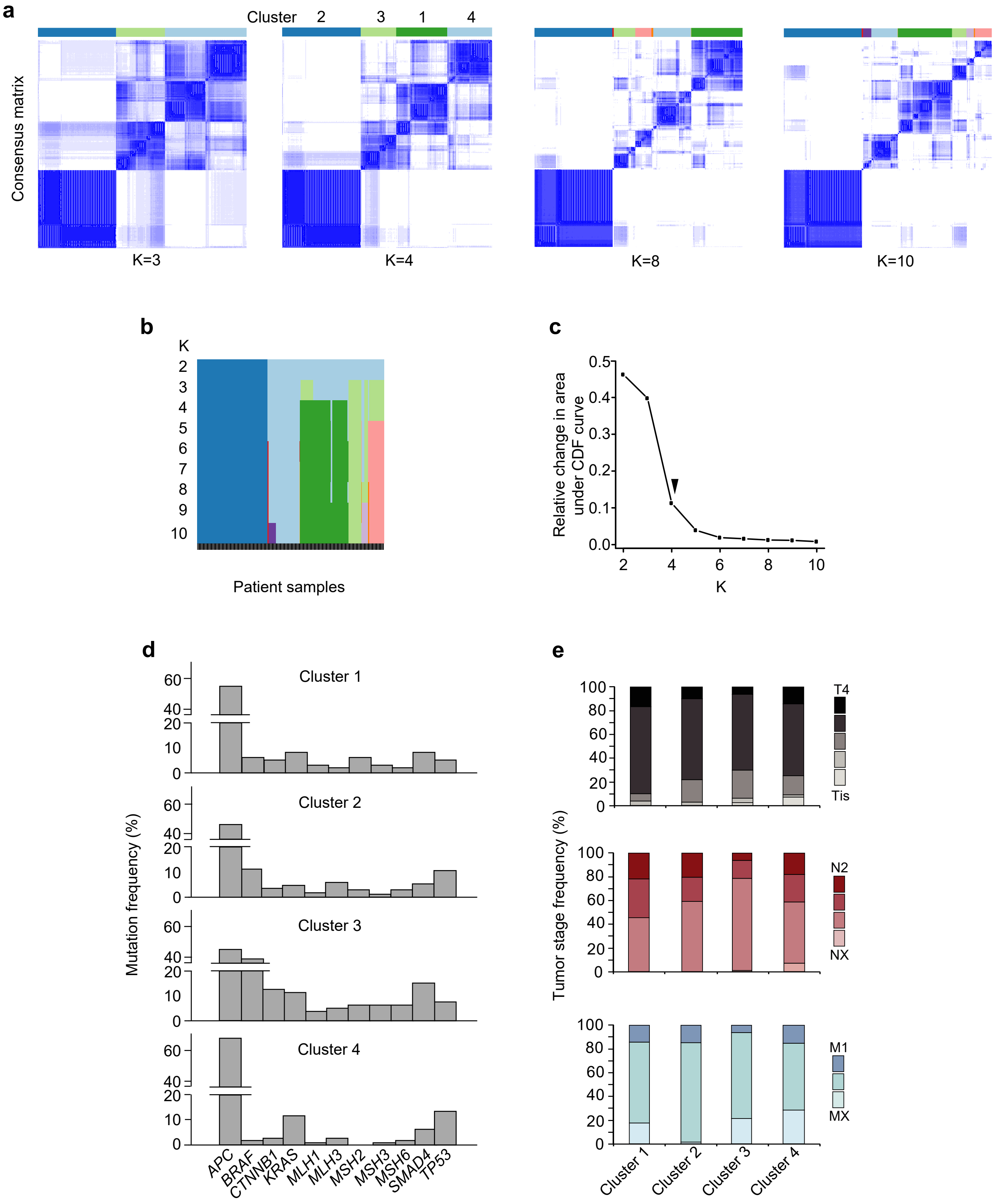

Supplementary Figure 1. Molecular, histologic and clinical characteristics of CRC subtypes. (**a**) Consensus clustering matrix of unsupervised classification for k=3, 4, 8 and 10. In each consensus matrix, both the rows and the columns were indexed with the same sample order and samples belonging to the same cluster are adjacent to each other. The consensus index for each pair of samples is shown in color from white (0%) to blue (100%). Colored boxes on the top of heatmap indicate a different cluster. (**b**) Tracking plot of k values in unsupervised classification. Bottom back rows: response to patient samples. Columns: response to k value, colors indicate different clusters. (**c**) Relative change area of Cumulative Distribution Function (CDF) curve for k= 2 to k= 10. At the black arrowhead, the gradient of the curve becomes less steep. Note that the curve tends to plateau after k=4, suggesting that the clustering is overfitting. (**d**) Frequency of the indicated gene mutations in each cluster. (**e**) Frequency of patient's TNM tumor stage for each Cluster.

Supplementary Figure 2

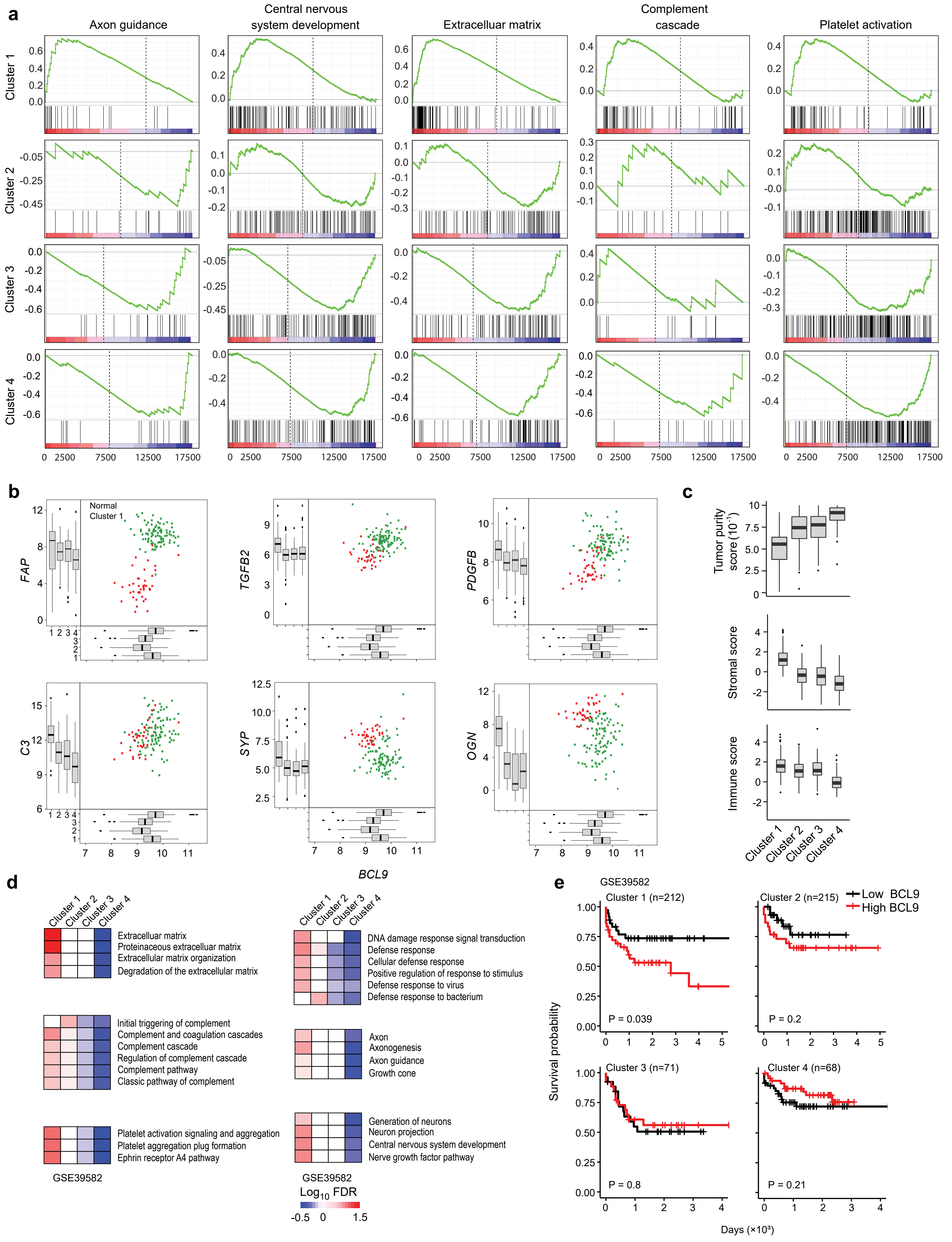

Supplementary Figure 2. Analyses of gene set enrichment and tumor purity of CRC subtypes. (**a**) Gene set enrichment analysis for the indicated cluster and gene function. For each indicated cluster signature genes were compared with the average expression of the other three clusters. (**b**) Expression analysis of BCL9 and the indicated genes in different Clusters. Red dots: normal colon epithelium, Green dots: CRC samples in Cluster 1. (**c**) Analysis of tumor cell signaling purity across Clusters. Cluster 1 displays the lower purity score and highest stromal cell score among all four Clusters. (**d**) GSEA of CRC clusters generated by GSE39582. Color-code response to log10 FDR level (red and blue: high and low confidence, respectively). (**e**) Kaplan-Meier plot of CRC survival probability according to GSE39582 cluster and BCL9 expression levels (high: top 25%; low: bottom 25%). Data are displayed as mean ±SD.

Supplementary Figure 3

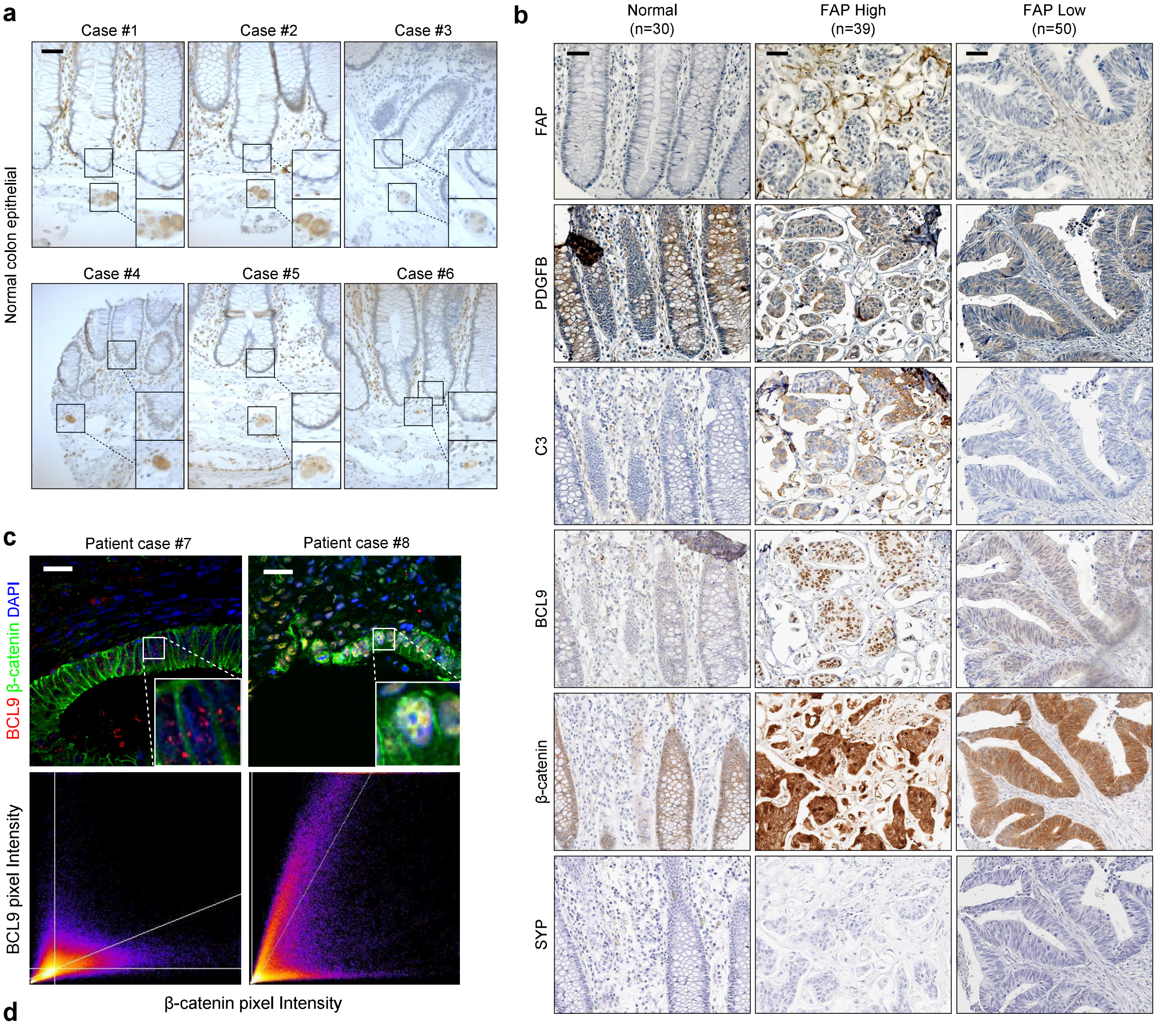

d

BCL9	β-catenin	FAP LOW	FAP HIGH
(+)	(+)	24%	36%
(+)	(-)	46%	41%
(-)	(+)	6%	10%
(-)	(-)	24%	13%

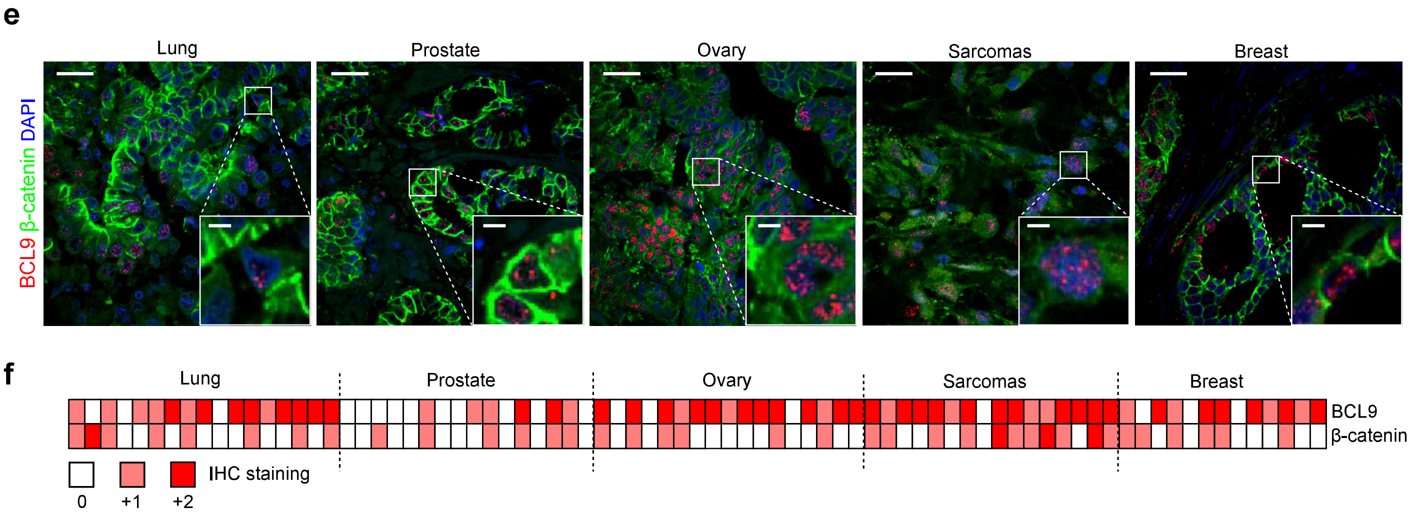

Supplementary Figure 3. Analyses of protein expression by immunohistochemistry and immunofluorescence. (**a**) Immunostains showing high expression in stromal and neuronal, but low expression of BCL9 in epithelial cells of normal colon mucosa. Scale bar: 10µm. (**b**) Representative IHC of the indicated proteins in serial consecutive sections from a tissue microarray containing 89 CRC and 30 normal colon mucosal samples in FAP low and high groups. Scale bar: 20µm. **(c)** Representative IF staining (top) and fluorescent colocalization threshold (bottom) of BCL9 and β-catenin in a tissue biopsy containing areas with inactivate (left) and active (right) β-catenin CRC patient samples. Correlation of pixels intensity was calculated using ImageJ, note colocalization of BCL9 and β-catenin was shown in case #8 but not case #7. (**d**) Table showing number of cases with BCL9 and/or β-catenin nuclear staining in FAP low and high groups. Representative IF (**e**) and heatmap (**f**) of BCL9 and β-catenin staining in a TMA containing the indicated cancer types. Numbers and color intensities indicate the intensity of nuclear staining; Red, 2: strong; Pink, 1: weak; White, 0: no staining. Scale bar: 20µm, 5µm.

Supplementary Figure 4

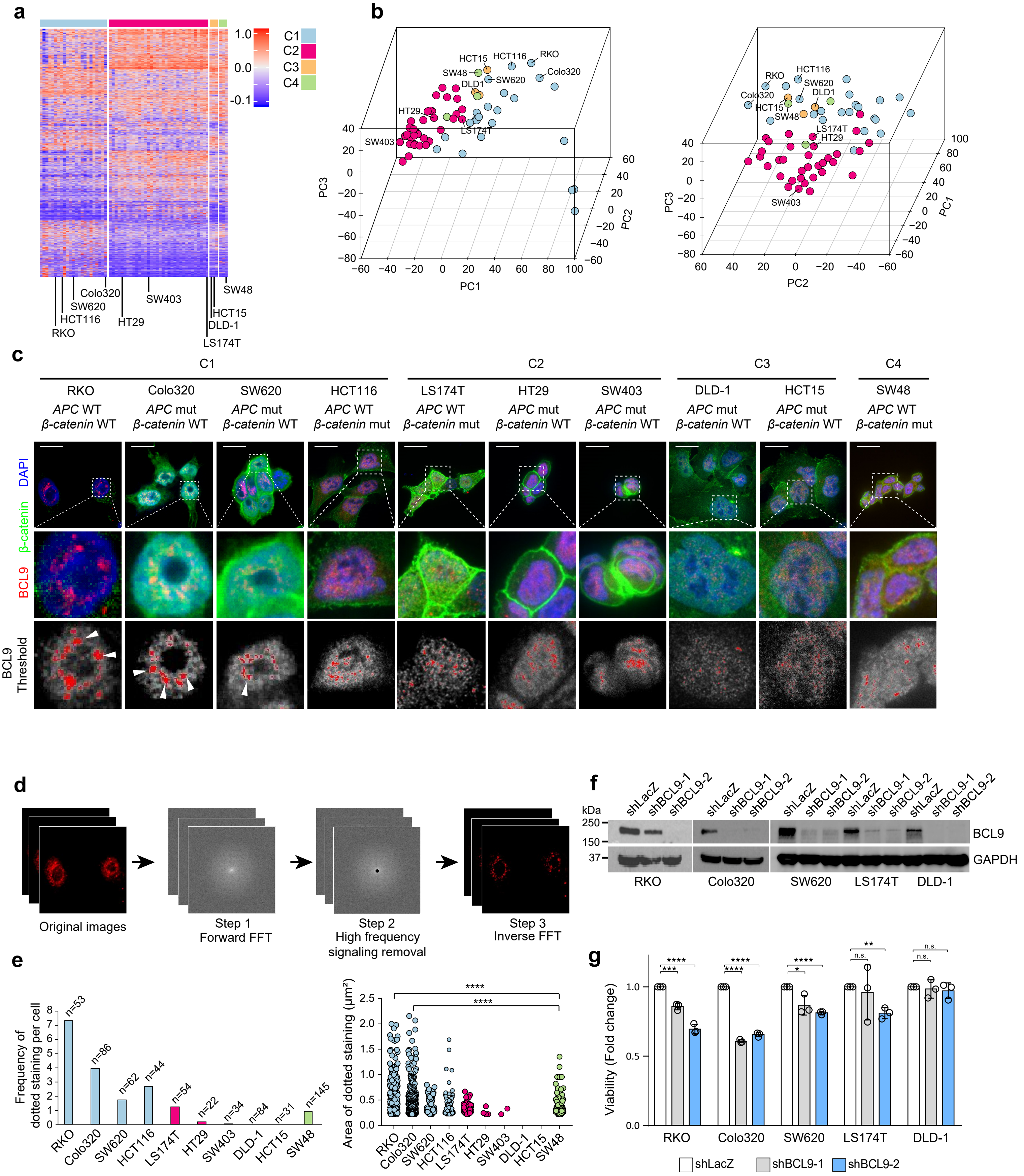

Supplementary Figure 4. Characterization of CRC cell lines by gene expression and BCL9 nuclear staining. (**a**) Gene expression profiling of CRC cell lines which represented different CRC clusters. (**b**) PCA analysis in two different perspective of CRC clustered cell lines. (**c**) IF of BCL9 and β-catenin in the indicated cell lines shown at low (top), high (middle) magnification, and highest 2% intensity of BCL9 staining (bottom). Scale bar: low magnification, 20µm; high magnification,1µm. (**d**) Schematic representation of BCL9 high frequency signaling removal. FFT: Fast Fourier Transform. (**e**) Frequency (left) and size area (right) plots of BCL9 punctate staining in the indicated cell lines. ****: P<1e-6; BCL9 immunoblots (IB) (**f**) and cell viability (**g**) in the indicated cell lines after lentiviral transduction with or shBCL9 or shLacZ used as controls. shBCL9-1 and shBCL9-2 indicate two different hairpins. P values were calculated using Student's *t* test. *: P<0.05; **: P<0.01; ***: P<0.001; ****: P<0.0001; n.s.: not significant. Data are displayed as mean ±SD.

Supplementary Figure 5

a

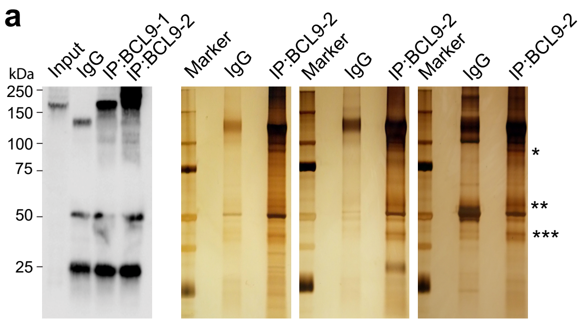

b

Name	Mean Total	Zone
VCP	77	*
SFPQ	22	*
HNRNPM	13	*
NONO	40	**
HNRNPK	33	**
PTBP1	8	**
RBMX	18	***
ILF2	15	***
ENO1	8	***

c

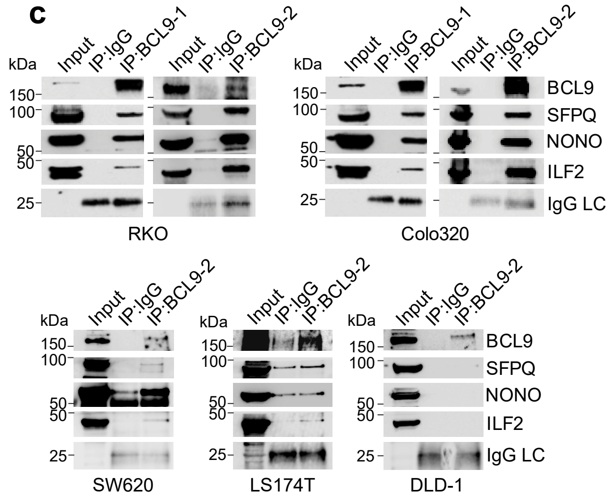

d

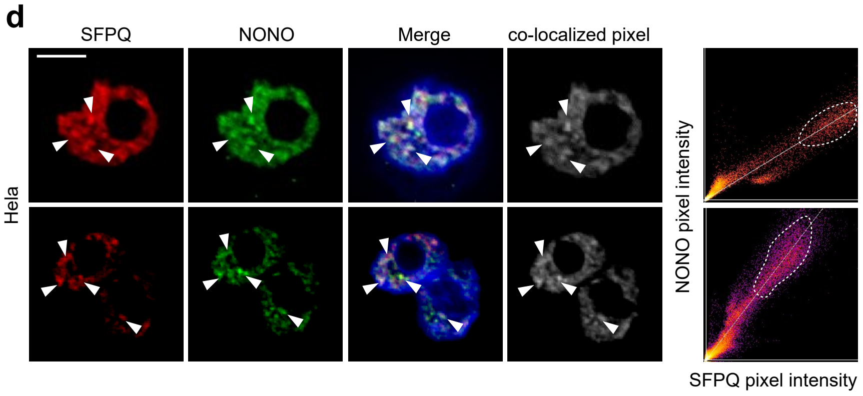

e

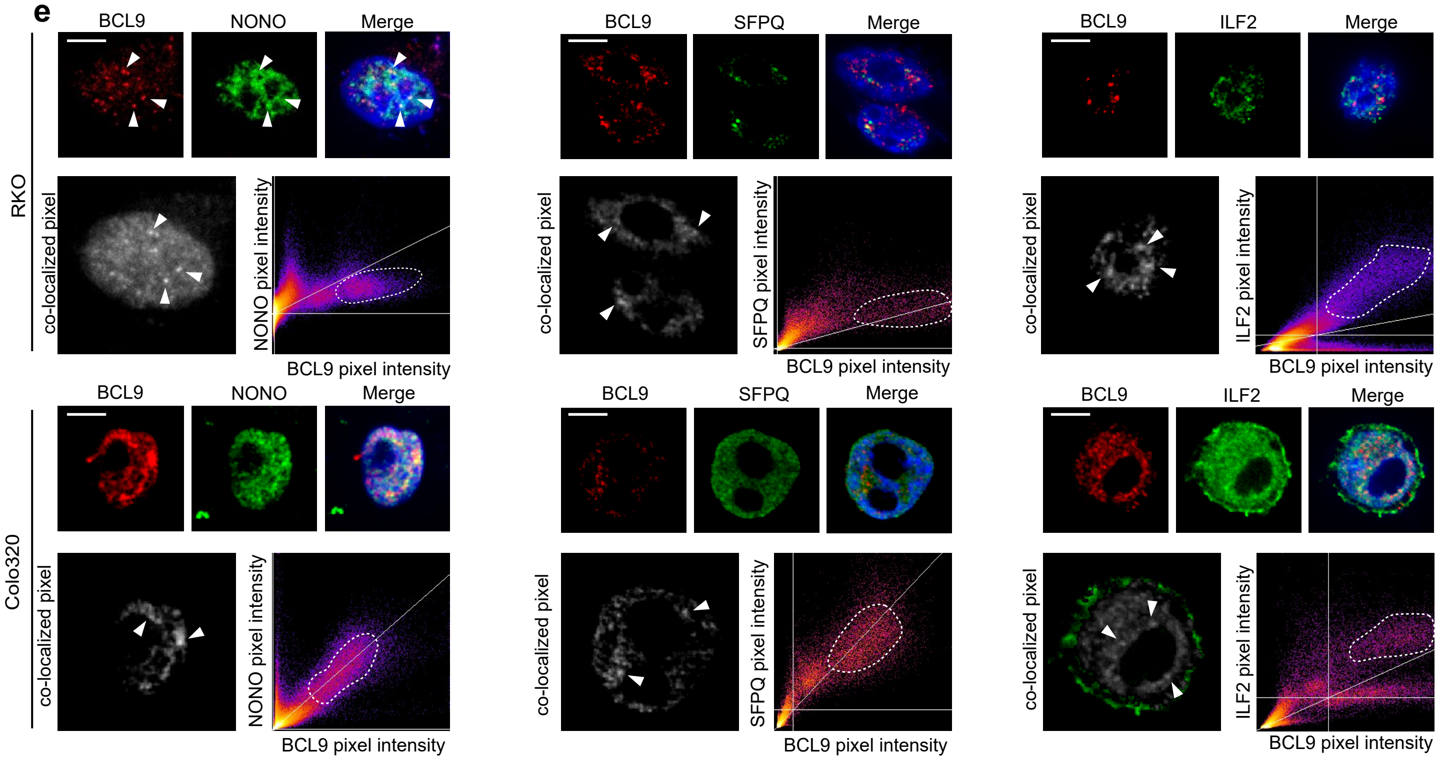

Supplementary Figure 5. Analyses of BCL9 interaction with paraspeckle proteins. (**a**) BCL9 IB (left) and silver stained gels (right) of immunoprecipitated proteins in Colo320 cells. *, **, and *** indicate bands present in anti-BCL9 but not in anti-IgG groups. **(b)** Table of protein names and the average of total peptide number for each of the bands indicated and excised from gels shown in "a" and analyzed by MS. (**c**) IP coupled IB identification of the indicated proteins and cell lines. BCL9-1 and BCL9-2 correspond to two different anti-BCL9 antibodies. (**d**) Representative IF of NONO and SFPQ in Hela cells. Colocalization threshold was calculated by using ImageJ, dotted areas represent a group of pixels that have high intensity for both NONO and SFPQ. Scale bar: 5 µm. (**e**) Representative IF of BCL9 colocalization with NONO (left), SFPQ (middle), and ILF2 (right) in RKO (top) and Colo320 (bottom) cells. Colocalization pixels and colocalization threshold are shown. Dotted areas represent a group of pixels which have high intensity for both BCL9 and indicated protein. Arrow heads indicate dotted areas with highest pixels for colocalization of both BCL9 and indicated proteins. Scale bar: 2µm.

Supplementary Figure 6

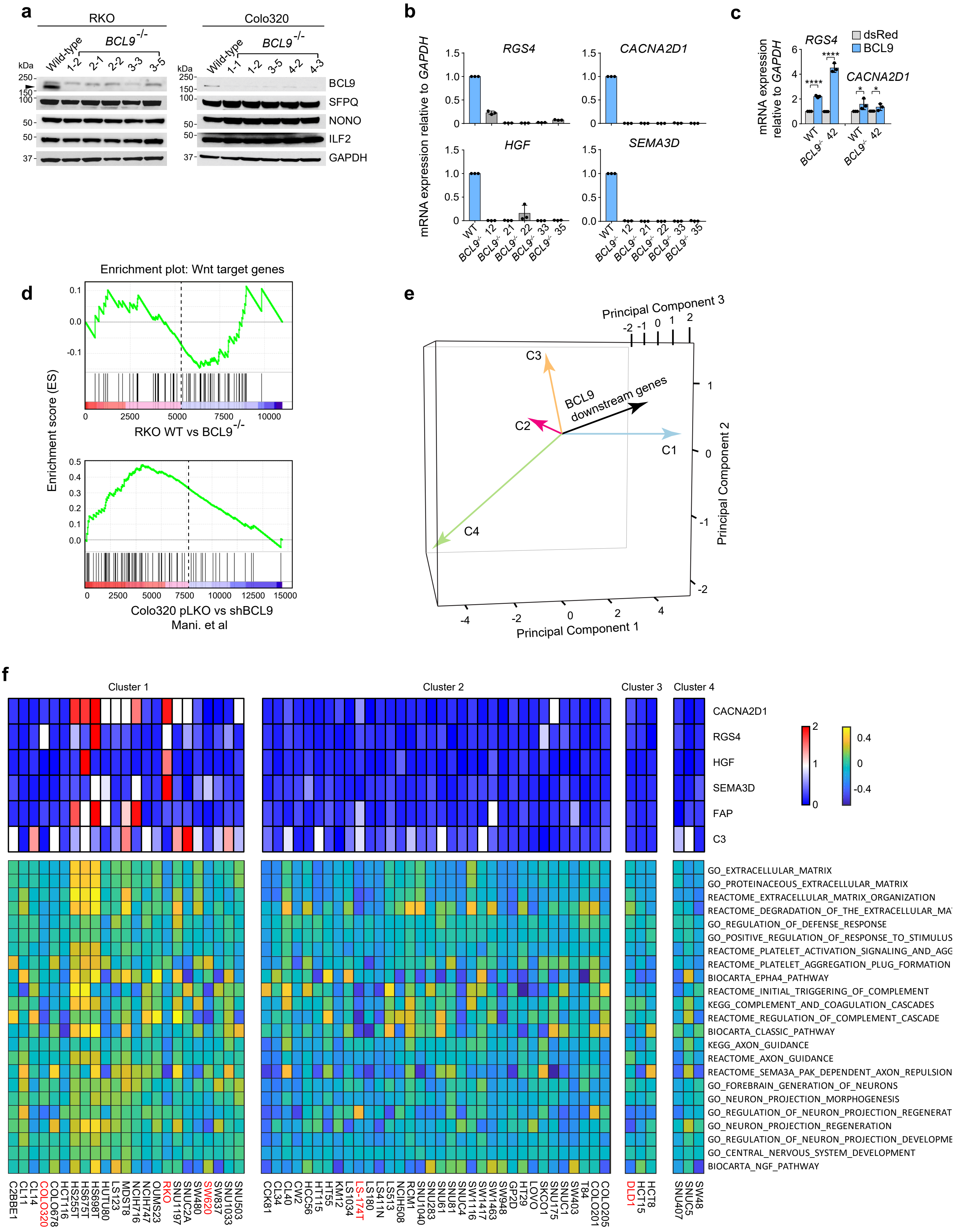

Supplementary Figure 6. Gene expression analyses in wild-type and BCL9 knockout CRC cell lines. (**a**) Expression of the indicated proteins and cell lines in wild-type and *BCL9* knockout clones. Numbers next to BCL9 indicate different knockout clones. RNA-seq results were verified by RT-qPCR in (**b**)five knockout clones and (**c**) one rescued clone. P values were calculated using Student's *t* test. *: P<0.05, ****: P<0.0001. (**d**) Gene set enrichment analysis of down-regulated genes in BCL9 deficient RKO (top) and Colo320 (bottom) cells. (**e**) PCA analysis of differentially expressed gene between wild-type and BCL9 knock out cells. (**f**) Heatmap of indicated gene expression (top) and pathway activation (bottom) in the indicated CRC cell lines. Data are displayed as mean ±SD.

Supplementary Figure 7

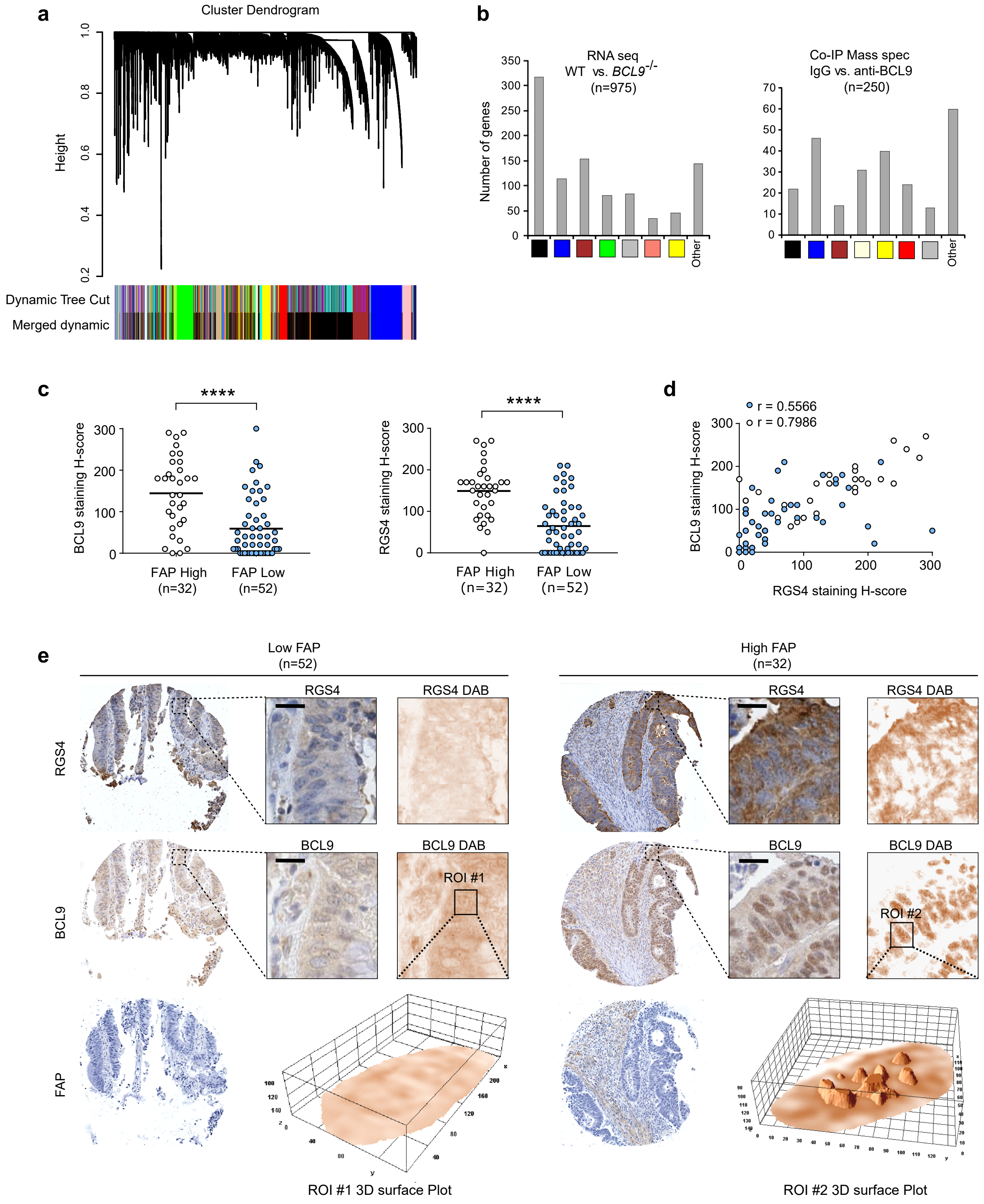

Supplementary Figure 7. BCL9 downstream genes form a co-regulation network that negatively correlates with prognosis. (**a**) Gene dendrogram obtained by average linkage hierarchical clustering of CRC Cluster 1. The colored row underneath the dendrogram shows the module assignment determined by Dynamic Tree Cut (upper) and merged dynamic (lower). (**b**) Distribution plots of downregulated genes in BCL9 knockout cells (left) and genes encoding BCL9-interacting proteins as per IP studies (right) in the transcriptional network matrix. (**c**) IHC H-scores of the indicated proteins in FAP low and high groups from a CRC TMA. The H-scores and FAP low and high groups were defined in material and methods. P values were calculated using Student's *t* test. ****: P<0.001; ns: not significant. (**d**) Correlation plots of IHC H-score of BCL9 and RGS4 in FAP low and high groups. (**e**) Representative IHC BCL9, RGS4 and FAP in FAP low and high groups; the staining intensity of the region of interest (ROI) was displayed by 3D surface plot. Scale bar: 10μm.

Supplementary Figure 8

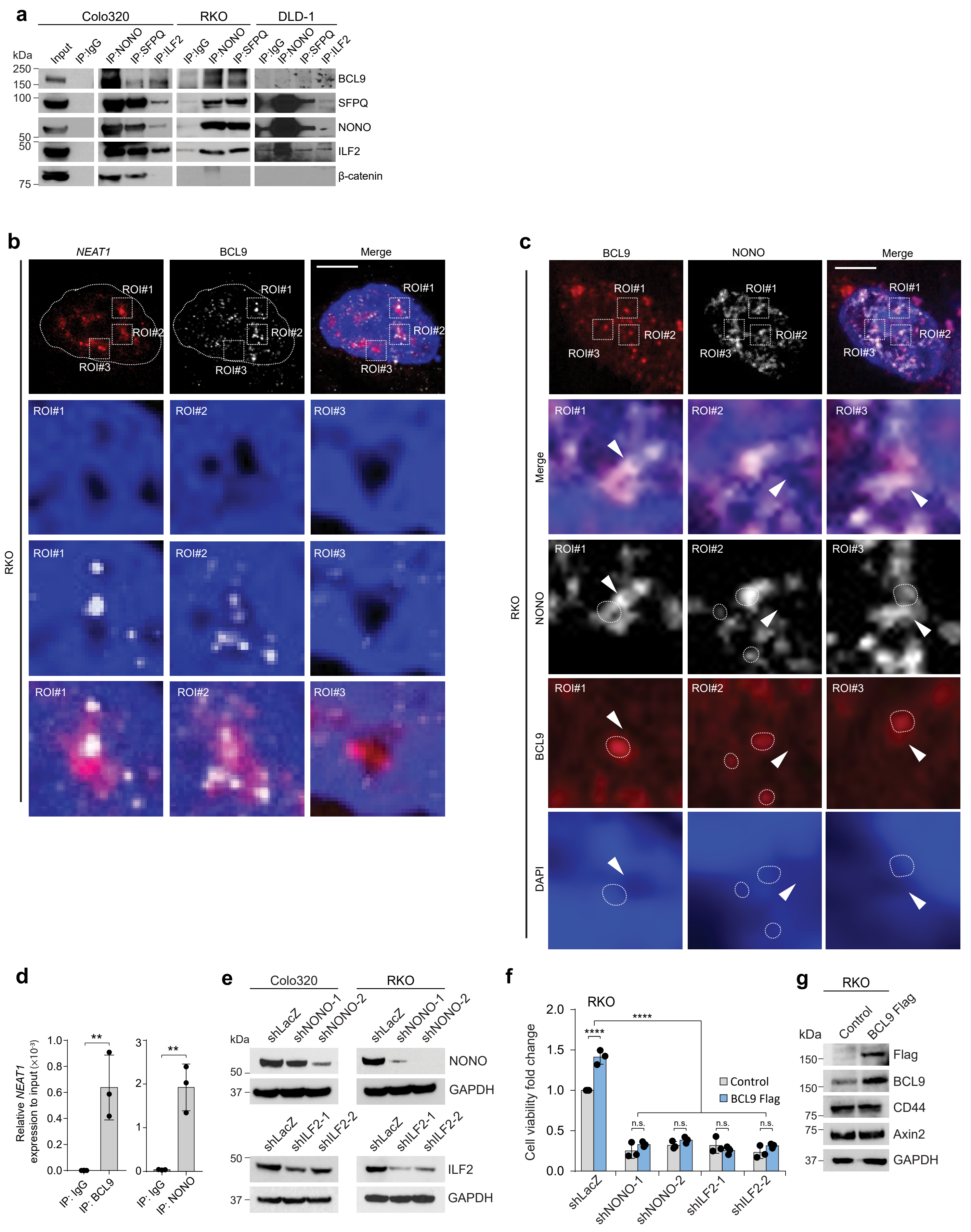

Supplementary Figure 8. Analyses of BCL9 interaction with paraspeckle proteins and localization within paraspeckles. (**a**) Co-IP with the indicated specific antibodies in the indicated CRC cells. (**b**) Combined BCL9 IF with *NEAT1* FISH in RKO cells. Dotted areas represent interchromosomal regions. (**c**) IF of NONO and BCL9 in RKO cells. Dotted areas indicate the position of BCL9 dotted staining. White arrow heads indicate interchromosomal region. ROI: region of interest. Scale bar: 5 μm. (**d**) RIP-PCR detected BCL9, NONO interaction with *NEAT1* non-coding RNA in RKO cells. P values were calculated using Student's *t* test. **: P<0.01. (**e**) Knockdown of NONO and ILF2 with two different shRNAs in RKO and Colo320 cell lines. (**f**) Cell viability of control and RKO cells overexpressing BCL9 after lentiviral transduction with shRNAs against LacZ, NONO or ILF2. P values were calculated using Student's *t* test. ****: P<0.0001; n.s.: not significant. shNONO-1, shNONO-2, shILF2-1 and shILF-2-2 indicate two different hairpins. (**g**) IB of Wnt/β-catenin downstream target genes in RKO cells overexpressing BCL9. Data are displayed as mean ±SD.

Supplementary Figure 9

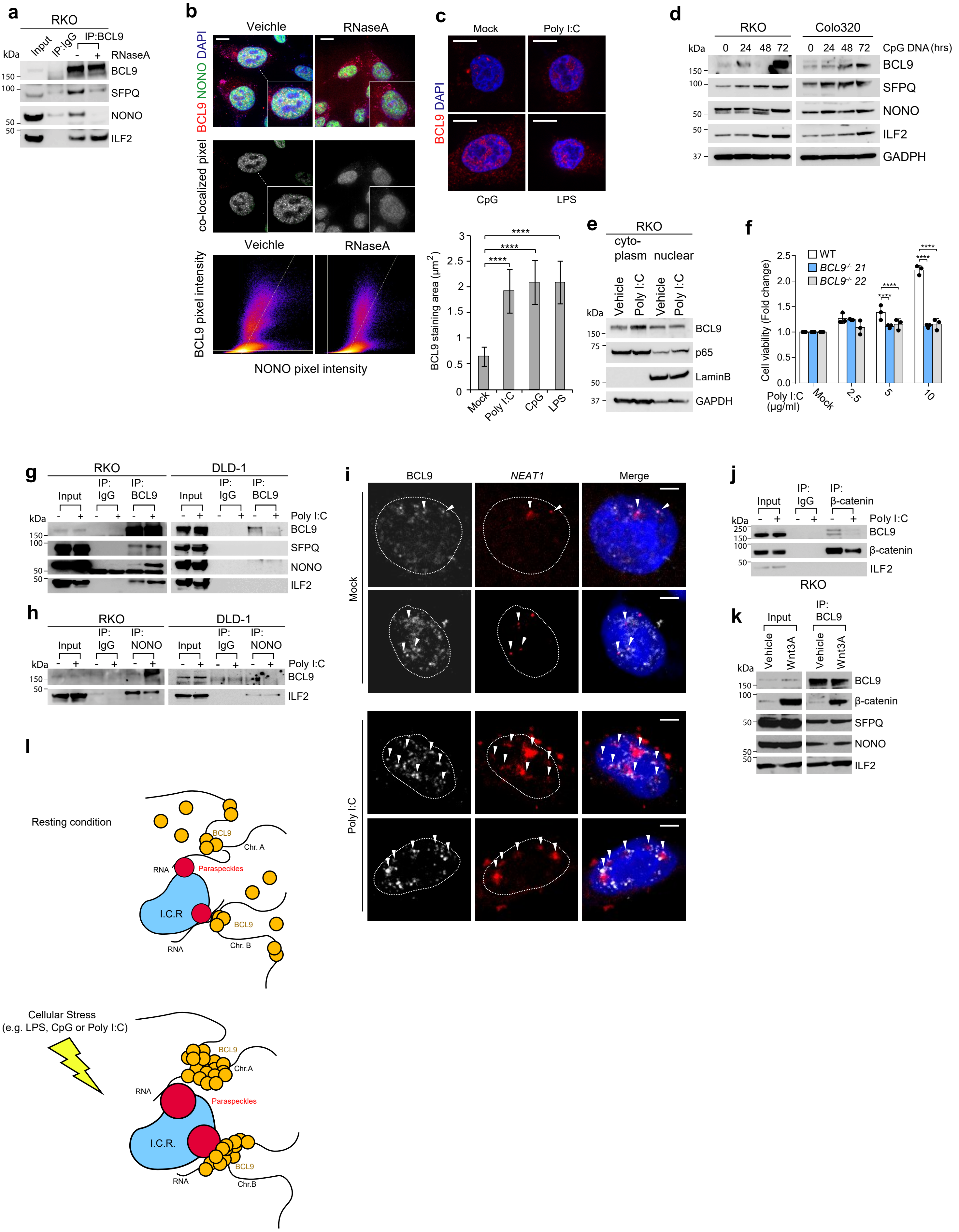

Supplementary Figure 9. BCL9 interaction with paraspeckle proteins and localization around paraspeckles. (**a**) Co-IP with BCL9 specific antibody in RKO cell lysates with or without RNase treatment. (**b**) Representative IF staining (top) and colocalization threshold (bottom) of BCL9 and NONO in control or RNase treated RKO cells. Scale bar: 10μm. (**c**) IF of BCL9 in RKO cells untreated or treated with Poly I:C, CpG DNA and LPS for 6hrs (top). BCL9 staining area as calculated by ImageJ2 (bottom). Scale bar: 2μm. P values were calculated using Student's *t* test. ****: P<0.0001. (**d**) Time course IB of the indicated protein in RKO and Colo320 cells treated in the absence or presence of CpG DNA. (**e**) IB of the indicated proteins in cytoplasmic and nuclear protein fractions of RKO cells untreated or treated with Poly I:C for 6hrs. (**f**) Cell viability analysis in wild-type and *BCL9* knockout RKO cells after treatment with Poly I: C. P values were calculated using Student's *t* test. ****: P<0. 0001. Numbers after $BCL9^{-/-}$ represent individual clones. (**g, h**) Co-IP with the indicated antibodies in untreated or Poly I:C treated indicated cells. (**i**) Combined BCL9 IF with *NEAT1* FISH in RKO cells treated or untreated with Poly I:C. White arrowhead indicate the *NEAT1* FISH signal, which is adjacent to and partially overlaps with BCL9 IF signal. Scale bar: 2μm. (**j**) Co-IP with anti-β-catenin antibody in untreated and poly I:C treated Colo320 cells. (**k**) Co-IP with anti-BCL9 antibody in untreated and Wnt3A treated RKO cells. (**l**) Schematic model for BCL9 interaction with paraspeckles. I.R.C represents interchromosomal regions. Data are displayed as mean ±SD.

Supplementary Figure 10

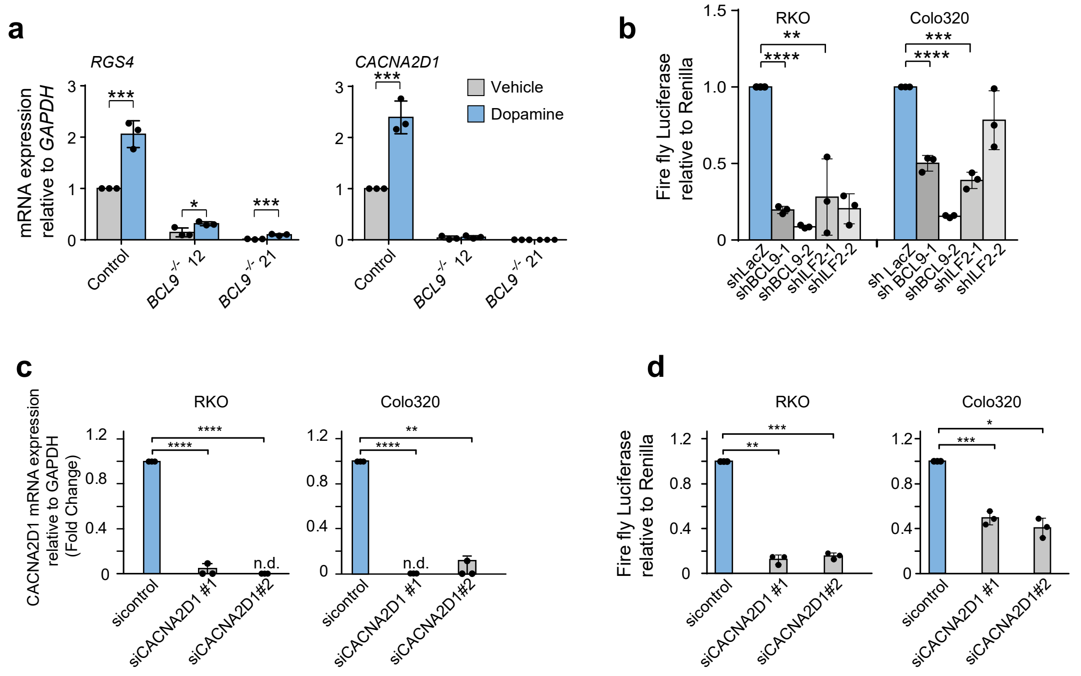

Supplementary Figure 10. Analyses of CACNA2D1 and RGS4 in RKO and Colo320 cells. (**a**) qRT-PCR analysis of RGS4 and CACNA2D1 mRNA expression in untreated or dopamine treated wild-type or *BCL9* knockout RKO cells. P values were calculated using Student's *t* test. *: P<0.05; ***: P<0.001. 12 and 21 represent different *BCL9* knockout clones. (**b**) NFATC2-dependent luciferase reporter activity in the indicated cells lentivirally transduced with either shLacZ, shBCL9, or shILF2. Numbers 1 and 2 indicate two different hairpins. P values were calculated using Student's *t* test. **: P<0.01, ***: P<0.001, ****: P<0.0001. (**c**) qRT-PCR analysis of *CACNA2D1* mRNA expression in indicated cell lines that have been treated with individual siRNAs. P values were calculated using Student's *t* test. **: P<0.01, ****: P<0.0001. (**d**) NFATC2-dependent luciferase reporter activity in the indicated cells transfected with siRNA of control or CACNA2D1. P values were calculated using Student's *t* test. *: P<0.05, **: P<0.01, ***: P<0.001. Data are displayed as mean ±SD.

Supplementary Figure 11

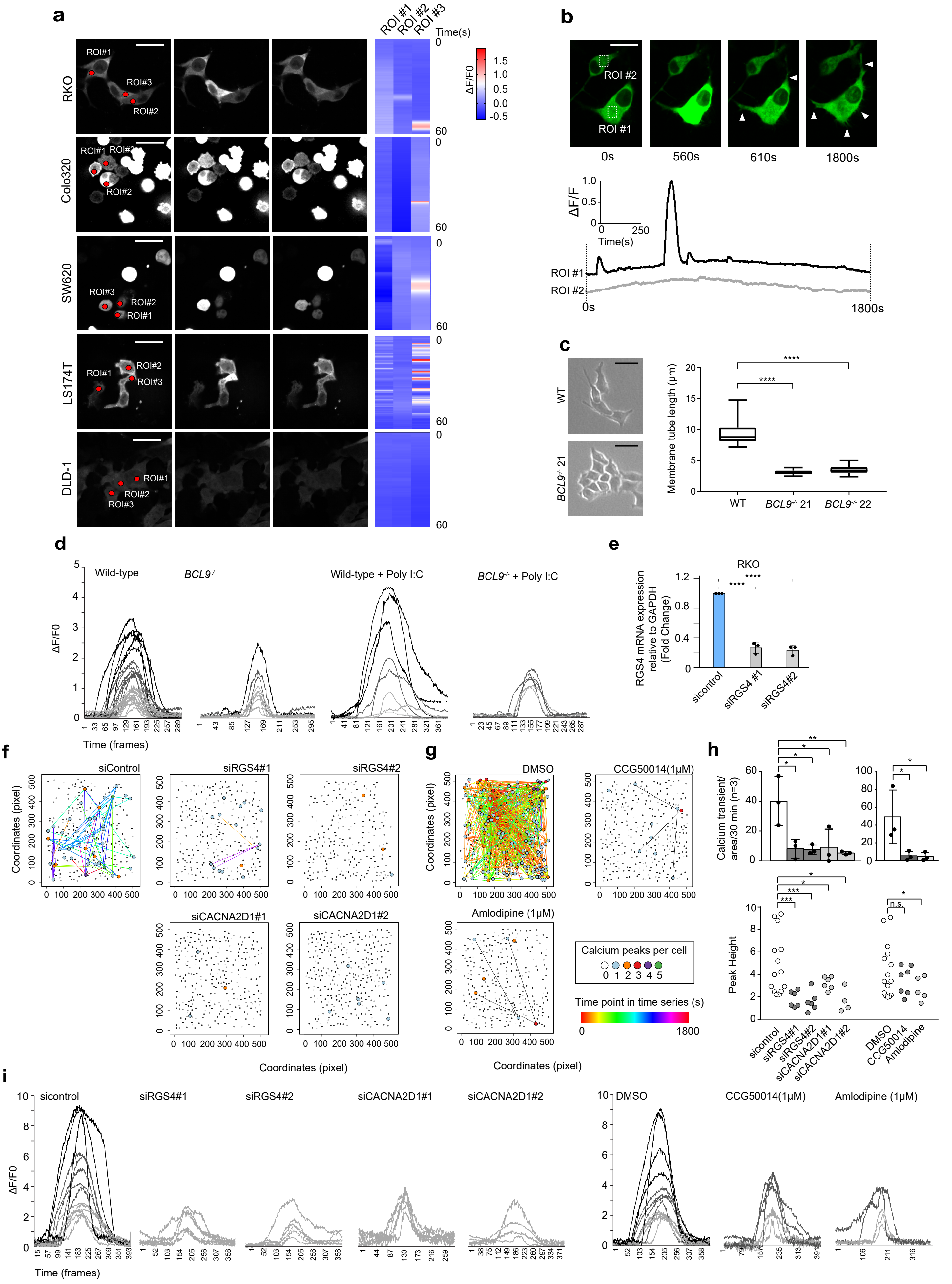

Supplementary Figure 11. Analyses of calcium waves in CRC cells. (**a**) Time-lapse imaging of calcium waves (left) and ΔF/F0 heatmap (right) of indicated region of interest (ROI, red dots) in indicated CRC cells. On the left, red color indicates waves pike, blue color represents baseline. Scale bar: 10μm. (**b**) Time-lapse imaging of calcium wave in indicated ROIs (top), ΔF/F0 (bottom) revealed that the extension of cell cytoplasm (white arrows) occurred after strenuous calcium transient (black line). Scale bar: 10μm. (**c**) Contrast phase imaging of wild-type and *BCL9* knockout RKO cells (left). The length of cytoplasmic extension was calculated by imageJ2 (right). P values were calculated using Student's *t* test. ****: P<0.001. Scale bar: 10μm. (**d**) Wavespike library of calcium waves in wild-type and *BCL9* knockout RKO cells in the absence or presence of Poly I: C. (**e**) mRNA expression fold changes in the RKO cells transfected with indicated siRNAs. P values were calculated using Student's *t* test. ****: P<0.0001. (**f**) Network of synchronized calcium transients in control and RGS4 or CACNA2D1 knockdown RKO cells. (**g**) Network of synchronized calcium transients in DMSO or CCG50014/Amlodipine treated RKO cells. Each dot represents one cell, the colored dots indicate the number of calcium wave events during 30 mins. Edged-linked dots represent synchronized calcium transients, colored edge indicates the timing of synchronized calcium transients. Data are displayed as mean ±SD for triplicate experiments and repeated twice. (**h**) Frequency (top), and peak height (bottom) of calcium transients in RKO cells treated with vehicle (control), siRNA or the indicated small molecule. P values were calculated using Student's *t* test. *: P<0.05; **: P<0.01; ***: P<0.001. n.s.: not significant. (**i**) Wavespike library of calcium waves in RKO cells treated with vehicle (control), siRNA or the indicated small molecule. Data are displayed as mean ±SD.

Supplementary Figure 12

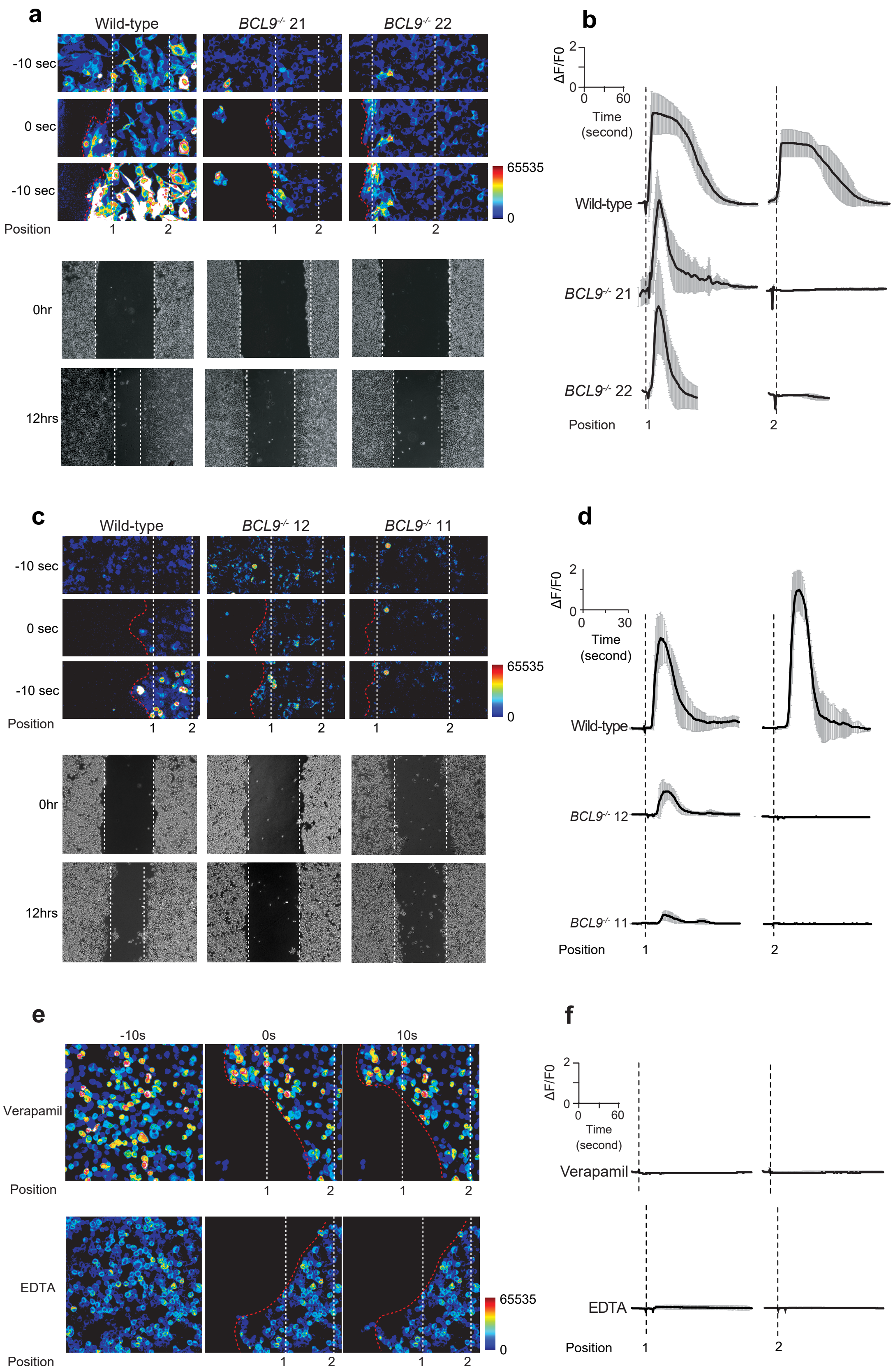

Supplementary Figure 12. Analysis of calcium waves using wound healing assay. (**a**) Time-lapse imaging of calcium wave spreading (top) and wound healing (bottom) at the indicated times after scratching of the monolayer surface in wild-type and *BCL9* knockout RKO cells. Top: Red dotted lines indicate the wound edge; numbered white dotted lines indicate two coordinates that are close (1) or distant (2) to the wound edge. Bottom: white dotted line indicates edges of the wound. Wound healing in *BCL9* knockout RKO cells is delayed after 12 hrs. Scale bar: 20μm. (**b**) ΔF/F0 in cells at position 1 and 2 in wild-type or BCL9 knockout Colo320 cells. (**c**) Time-lapse imaging of calcium wave spreading (top) and wound healing (bottom) at the indicated times after scratching of monolayer surface in wild-type and BCL9 knockout Colo320 cells. Top: Red dotted lines indicate the wound edge; numbered white dotted lines Indicate two coordinates that are close (1) or distant (2) to the wound edge. Bottom: white dotted line indicates edges of the wound. Scale bar: 20μm. (**d**) ΔF/F0 in cells at position 1 and 2 in wild-type or BCL9 knockout Colo320 cells. (**e**) Time-lapse imaging revealed that the calcium wave disappeared after scratching monolayer surface in EDTA or verapamil treated RKO cells. Scale bar: 20μm. (**f**) ΔF/F0 in cells at position 1 and 2 in EDTA and verapamil treatment RKO cells.

Supplementary Figure 13

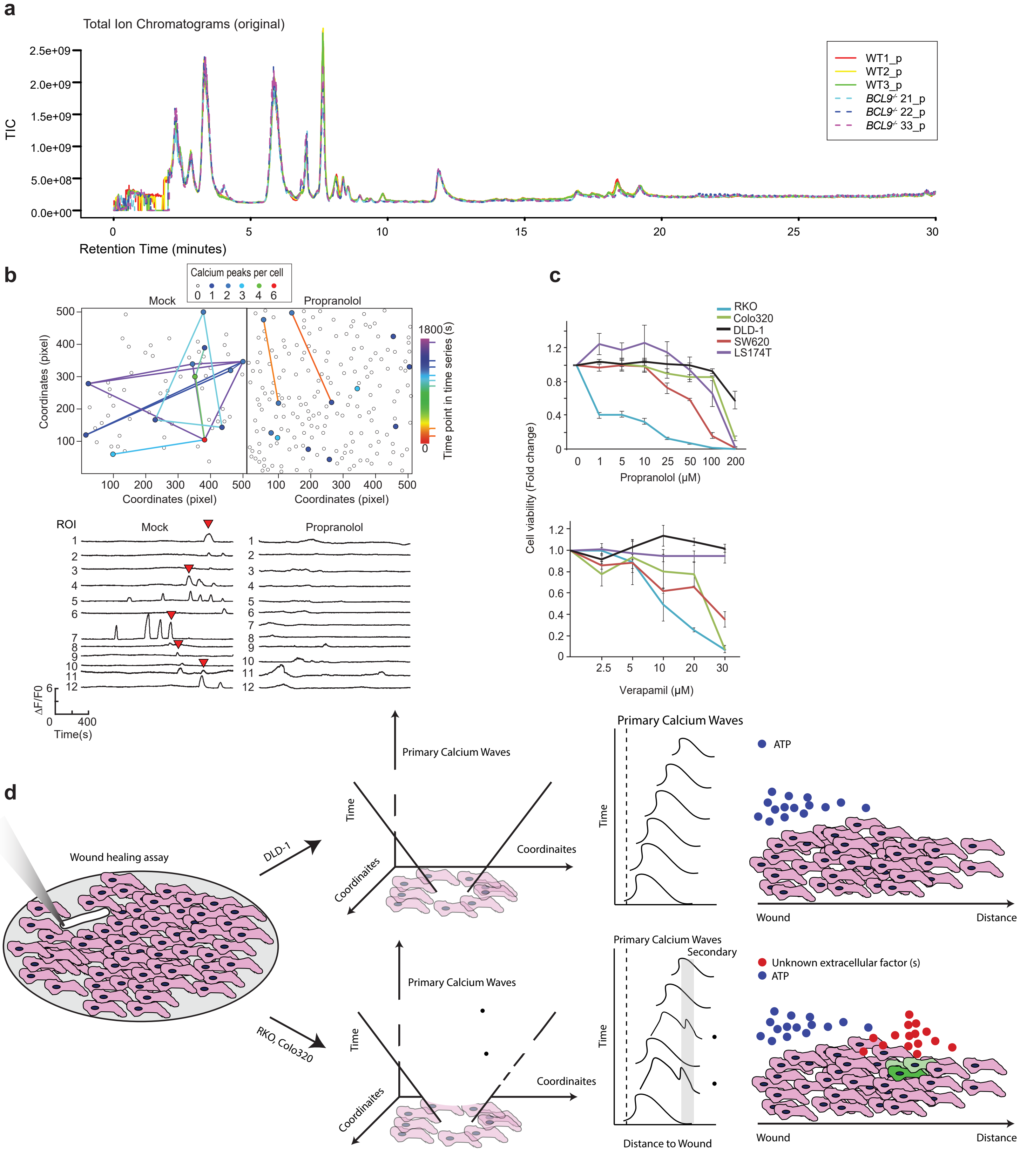

Supplementary Figure 13. Cell viability in CRC cells treated with Propranolol or Verapamil. (**a**) Total Ion Chromatograms of CM from wild type and *BCL9* knockout RKO cells (n=3). (**b**) Network of calcium transients spreading and ΔF/F0 (top) of synchronized calcium waves in control and propranolol treated RKO cells. (**c**) Cell viability of indicated CRC cell lines treated in the absence or increased concentrations of propranolol (top) or verapamil (bottom). (**d**) Model of calcium wave spreading in Colo320, RKO and DLD-1 cells. Data are displayed as mean ±SD.

Supplementary Table 1. List of PCR primers.

Gene	Sequence	Ref.
CACNA2D1	Forward CTGACGGTCCAAATCCTTGT	This paper
	Reverse: TGCCAGATACCAGCCAAAGT	
RGS4	Forward CCAGAGAGTGAGCCAAGAGG	This paper
	Reverse ATCTTTTTGGCCTTGGGACT	
HGF	Forward CATGTCCTCCTGCATCTCCT	This paper
	Reverse AGCCTTGCAAGTGAATGGAA	
SEMA3D	Forward GCATCGAGAGGAGTTGAAGC	This paper
	Reverse GCCCTCTGGGTATTTTCCAT	
TGF-β2	Forward ATTGCTGCCTACGTCCACTT	This paper
	Reverse TTGGGTGTTTTGCCAATGTA	
IL-10	Forward TCCCTGTGAAAACAAGAGCA	This paper
	Reverse ATAGAGTCGCCACCCTGATG	
NEAT1	Forward GATCTTTTCCACCCCAAGAGTACATAA	Imamura K *et al.* Mol Cell. 2014 Feb
	Reverse CTCACACAAACACAGATTCCACAAC	
