# Supplementary material for: BCL9 provides multi-cellular communication properties in colorectal cancer by interacting with paraspeckle proteins
Source: Nat Commun. 2020 Jan 7;11:19. doi: 10.1038/s41467-019-13842-7 (PMC6946813; doi:10.1038/s41467-019-13842-7)
Supplement: Supplementary file 2 — Description of Additional Supplementary Files [file 41467_2019_13842_MOESM2_ESM.pdf]

Description of Additional Supplementary Files

Title: Supplementary Data 1. List of Cluster 1 specific-upregulated genes.

Title: Supplementary Data 2. Immunoprecipitation coupled mass spec analysis identified BCL9 interaction proteins.

Title: Supplementary Data 3. Differentially expressed genes between wild type and BCL9 knockout RKO cells.

Title: Supplementary Movie 1: Spontaneous calcium transients in colorectal cancer cell lines.
