# Supplementary material for: BCL9 provides multi-cellular communication properties in colorectal cancer by interacting with paraspeckle proteins
Source: Nat Commun. 2020 Jan 7;11:19. doi: 10.1038/s41467-019-13842-7 (PMC6946813; doi:10.1038/s41467-019-13842-7)
Supplement: Supplementary file 7 — Reporting Summary [file 41467_2019_13842_MOESM7_ESM.pdf]

nature research

Corresponding author(s): Ruben Carrasco,Associate Professor of Pathology, Harvard Medical School,Dana-Farber Cancer Institute, Department of Medical Oncology

Last updated by author(s): Oct 29, 2019

Reporting Summary

Nature Research wishes to improve the reproducibility of the work that we publish. This form provides structure for consistency and transparency in reporting. For further information on Nature Research policies, seeAuthors & Referees and theEditorial Policy Checklist .

Statistics

For all statistical analyses, confirm that the following items are present in the figure legend, table legend, main text, or Methods section.

n/a	Confirmed	
☐	☒	The exact sample size (*n*) for each experimental group/condition, given as a discrete number and unit of measurement
☐	☒	A statement on whether measurements were taken from distinct samples or whether the same sample was measured repeatedly
☐	☒	The statistical test(s) used AND whether they are one- or two-sided<br>*Only common tests should be described solely by name; describe more complex techniques in the Methods section.*
☒	☐	A description of all covariates tested
☒	☐	A description of any assumptions or corrections, such as tests of normality and adjustment for multiple comparisons
☐	☒	A full description of the statistical parameters including central tendency (e.g. means) or other basic estimates (e.g. regression coefficient) AND variation (e.g. standard deviation) or associated estimates of uncertainty (e.g. confidence intervals)
☐	☒	For null hypothesis testing, the test statistic (e.g. *F*, *t*, *r*) with confidence intervals, effect sizes, degrees of freedom and *P* value noted<br>*Give P values as exact values whenever suitable.*
☒	☐	For Bayesian analysis, information on the choice of priors and Markov chain Monte Carlo settings
☐	☒	For hierarchical and complex designs, identification of the appropriate level for tests and full reporting of outcomes
☐	☒	Estimates of effect sizes (e.g. Cohen's *d*, Pearson's *r*), indicating how they were calculated

Our web collection on statistics for biologists contains articles on many of the points above.

Software and code

Policy information about availability of computer code

Data collection	The RNA-seq data of colorectal cancer patients was downloaded from TCGA (https://cancergenome.nih.gov/)
Data analysis	Global Synchronization Index was calculated by FluoroSNNAP. Correlation Matrix was calculated by WCGNA R package. fluorescence intensity was calculated by imagej2.

For manuscripts utilizing custom algorithms or software that are central to the research but not yet described in published literature, software must be made available to editors/reviewers. We strongly encourage code deposition in a community repository (e.g. GitHub). See the Nature Research guidelines for submitting code & software for further information.

Data

Policy information about availability of data

All manuscripts must include a data availability statement. This statement should provide the following information, where applicable:

- Accession codes, unique identifiers, or web links for publicly available datasets
- A list of figures that have associated raw data
- A description of any restrictions on data availability

The source data underlying Figs. 3e-f, 3h-l, 4h, 5a and 5h, and Supplementary Figs. 4f-g, 5a, 5c, 6a-c, 8a, 8d-g, 9a, 9e-h, 9j-k, 10a-d, 11e and 13c are provided as a source data file.

Field-specific reporting

Please select the one below that is the best fit for your research. If you are not sure, read the appropriate sections before making your selection.

Life sciences study design

All studies must disclose on these points even when the disclosure is negative.

Sample size	No statistical methods were used to predetermine sample size. Experiments were performed three times independently unless indicated. In previous studies using related experiments, the sample size has been determined to be sufficient to ensure reproducibility.
Data exclusions	No data exclusions
Replication	all attempts at replication were successful
Randomization	NA
Blinding	NA

Reporting for specific materials, systems and methods

We require information from authors about some types of materials, experimental systems and methods used in many studies. Here, indicate whether each material, system or method listed is relevant to your study. If you are not sure if a list item applies to your research, read the appropriate section before selecting a response.

Materials & experimental systems

n/a	Involved in the study	
☐	☒	Antibodies
☐	☒	Eukaryotic cell lines
☒	☐	Palaeontology
☐	☒	Animals and other organisms
☒	☐	Human research participants
☒	☐	Clinical data

Methods

n/a	Involved in the study	
☒	☐	ChIP-seq
☒	☐	Flow cytometry
☒	☐	MRI-based neuroimaging

Antibodies

Antibodies used

Rabbit polyclonal anti-BCL9 Abcam Cat# 37305
Rabbit polyclonal anti-BCL9 Mala Mani, et al., 2009 NA
Mouse monoclonal anti-BCL9 Abnova Cat# H00000607-M01
Rabbit polyclonal anti-β-catenin Cell signaling Cat# 9562L
Mouse monoclonal anti- β-catenin BD Transduction Laboratories Cat# 610154
Rabbit polyclonal anti-NONO Abcam Cat# ab70335
Mouse monoclonal anti-NONO Origene Cat# TA504777
Rabbit polyclonal anti-SFPQ Abcam Cat# ab38148
Mouse monoclonal anti-SFPQ ThermoFisher Cat# MA1-25325
Goat polyclonal anti-ILF2 ThermoFisher Cat# PA5-18718
Rabbit polyclonal anti-ILF2 Abnova Cat# H00003608-D01
Rabbit polyclonal anti-C3 Sigma Cat# HPA003563
Sheep polyclonal anti-FAP R&D Cat# AF3715
Rabbit polyclonal anti-PDGFB Abcam Cat# ab23914
Mouse monoclonal anti-SYP Lecia Cat# PA0299
Mouse monoclonal anti-RGS4 Santa Cruz Cat# sc-398348
Rabbit monoclonal anti-p65 Cell signaling Cat# 8242
Rabbit polyclonal anti-TLR3 Abcam Cat# ab62566
Mouse monoclonal anti-Flag Sigma Cat# A8592
Rabbit monoclonal anti-Axin2 Cell signaling Cat# 2151
Mouse monoclonal anti-CD44 Cell signaling Cat# 3570
Rabbit polyclonal anti-GAPDH Abcam Cat# ab9485
Goat polyclonal anti-Lamin-B1 Santa Cruz Cat# sc-6216
Normal mouse IgG Santa Cruz Cat# sc-2025
Normal rabbit IgG Santa Cruz Cat# sc-3888
Goat anti-rabbit IgG HRP linked Cell signaling Cat# 7074
Goat anti-mouse IgG HRP linked Cell signaling Cat# 7076
Donkey anti-goat IgG-HRP Santa Cruz Cat# sc-2020
Goat anti-mouse IgG Alexa Fluor 488 ThermoFisher Cat# A-11029

	Goat anti-rabbit IgG Alexa Fluor 546 ThermoFisher Cat# A-11035<br>Donkey anti-goat IgG Alexa Fluor 647 ThermoFisher Cat# A-21447
Validation	Antibodies were validated by the manufacturer with provided datasheets.

Eukaryotic cell lines

Policy information about cell lines

Cell line source(s)	all cell lines are purchased from ATCC
Authentication	Authenticated by the short tandem repeat (STR) DNA method at the Human Cell line Identity Verification facility at Dana-Farber Cancer Institute or using in our own lab the Promega Power Plex 16HS Kit.
Mycoplasma contamination	Mycoplasma contamination using e-Myco, a Mycoplasma PCR Detection Kit (Abbott).
Commonly misidentified lines<br>(See ICLAC register)	NA

Animals and other organisms

Policy information about studies involving animals; ARRIVE guidelines recommended for reporting animal research

Laboratory animals	6 weeks female CB17.Cg-PrkdcscidLystbg-J/Crl (Beige) mice was used for generating xenograft model in this study
Wild animals	NA
Field-collected samples	NA
Ethics oversight	All animal studies were conducted in accordance with the guidelines of and approved by the Animal Research Ethics Board of Dana Farber cancer institute

Note that full information on the approval of the study protocol must also be provided in the manuscript.
